# Differential Roles for Inner Membrane Complex Proteins across *Toxoplasma gondii* and *Sarcocystis neurona* Development

**DOI:** 10.1128/mSphere.00409-17

**Published:** 2017-10-18

**Authors:** Rashmi Dubey, Brooke Harrison, Sriveny Dangoudoubiyam, Giulia Bandini, Katherine Cheng, Aziz Kosber, Carolina Agop-Nersesian, Daniel K. Howe, John Samuelson, David J. P. Ferguson, Marc-Jan Gubbels

**Affiliations:** a Biology Department, Boston College, Chestnut Hill, Massachusetts, USA; b Gluck Equine Research Center, Department of Veterinary Science, University of Kentucky, Lexington, Kentucky, USA; c Department of Molecular and Cell Biology, Henry M. Goldman School of Dental Medicine, Boston University, Boston, Massachusetts, USA; d Nuffield Department of Pathology, Oxford University, John Radcliffe Hospital, Oxford, United Kingdom; Carnegie Mellon University

**Keywords:** IMC, *Sarcocystis*, *Toxoplasma*, endodyogeny, endopolygeny, gametocyte, merozoite, sporozoite

## Abstract

The inner membrane complex (IMC) of apicomplexan parasites contains a network of intermediate filament-like proteins. The 14 alveolin domain-containing IMC proteins in *Toxoplasma gondii* fall into different groups defined by their distinct spatiotemporal dynamics during the internal budding process of tachyzoites. Here, we analyzed representatives of different IMC protein groups across all stages of the *Toxoplasma* life cycle and during *Sarcocystis neurona* asexual development. We found that across asexually dividing *Toxoplasma* stages, IMC7 is present exclusively in the mother’s cytoskeleton, whereas IMC1 and IMC3 are both present in mother and daughter cytoskeletons (IMC3 is strongly enriched in daughter buds). In developing macro- and microgametocytes, IMC1 and -3 are absent, whereas IMC7 is lost in early microgametocytes but retained in macrogametocytes until late in their development. We found no roles for IMC proteins during meiosis and sporoblast formation. However, we observed that IMC1 and IMC3, but not IMC7, are present in sporozoites. Although the spatiotemporal pattern of IMC15 and IMC3 suggests orthologous functions in *Sarcocystis*, IMC7 may have functionally diverged in *Sarcocystis* merozoites. To functionally characterize IMC proteins, we knocked out IMC7, -12, -14, and -15 in *Toxoplasma*. IMC14 and -15 appear to be involved in switching between endodyogeny and endopolygeny. In addition, IMC7, -12, and -14, which are all recruited to the cytoskeleton outside cytokinesis, are critical for the structural integrity of extracellular tachyzoites. Altogether, stage- and development-specific roles for IMC proteins can be discerned, suggesting different niches for each IMC protein across the entire life cycle.

**IMPORTANCE** The inner membrane complex (IMC) is a defining feature of apicomplexan parasites key to both their motility and unique cell division. To provide further insights into the IMC, we analyzed the dynamics and functions of representative alveolin domain-containing IMC proteins across developmental stages. Our work shows universal but distinct roles for IMC1, -3, and -7 during *Toxoplasma* asexual division but more specialized functions for these proteins during gametogenesis. In addition, we find that IMC15 is involved in daughter formation in both *Toxoplasma* and *Sarcocystis*. IMC14 and IMC15 function in limiting the number of *Toxoplasma* offspring per division. Furthermore, IMC7, -12, and -14, which are recruited in the G_1_ cell cycle stage, are required for stress resistance of extracellular tachyzoites. Thus, although the roles of the different IMC proteins appear to overlap, stage- and development-specific behaviors indicate that their functions are uniquely tailored to each life stage requirement.

## Abstract (precis)

The inner membrane complex (IMC) is a defining feature of apicomplexan parasites key to both their motility and unique cell division. To provide further insights into the IMC, we analyzed the dynamics and functions of representative alveolin domain-containing IMC proteins across developmental stages. Our work shows universal but distinct roles for IMC1, -3, and -7 during *Toxoplasma* asexual division but more specialized functions for these proteins during gametogenesis. In addition, we find that IMC15 is involved in daughter formation in both *Toxoplasma* and *Sarcocystis* tachyzoites, bradyzoites, and sporozoites. IMC14 and IMC15 function in limiting the number of *Toxoplasma* offspring per division. Furthermore, IMC7, -12, and -14, which are recruited in the G_1_ cell cycle stage, are required for stress resistance of extracellular tachyzoites. Thus, although the roles of the different IMC proteins appear to overlap, stage- and development-specific behaviors indicate that their functions are uniquely tailored to each life stage requirement.

## INTRODUCTION

*Toxoplasma gondii* is an obligate intracellular parasite causing opportunistic infection in humans (1). *Toxoplasma* is a member of the phylum *Apicomplexa*, consisting of approximately 5,000 species that are nearly all obligate intracellular parasites and are defined by the presence of a complex apical cytoskeleton and secretory organelles (2). Other pathogens in this phylum of medical and economic importance include *Plasmodium*, the causative agent of malaria, and *Cryptosporidium*, causing severe enteritis, whereas *Eimeria*, *Neospora*, *Babesia*, and *Sarcocystis* are of veterinary importance. The *Toxoplasma* life cycle comprises all warm-blooded vertebrates, including humans, as intermediate hosts, whereas the sexual (coccidian) cycle is restricted to felids, which serve as the definitive host. *Sarcocystis neurona* and *Toxoplasma* are closely related cyst-forming coccidia; both form tissue cysts in their intermediate hosts, which for *S. neurona* are scavengers (e.g., raccoons, armadillos, skunks), with the opossum as the definitive host. *S. neurona* causes equine protozoal myeloencephalitis upon accidental horse infection (3).

Apicomplexan life cycles are composed of a series of sequential development stages, several of which undergo distinct division modes in different hosts. In the intermediate host, the *Toxoplasma* tachyzoite divides by endodyogeny, an internal budding process forming two daughter parasites per division round (4). *S. neurona* merozoites in the intermediate host also divide by internal budding; however, they form up to 64 daughters per division round from a large polyploid nucleus, resulting of several rounds of S phase without karyokinesis or cytokinesis (the large polyploid mother cell is called a schizont; the division process is called endopolygeny) (5, 6). Coincident with a mounting immune response, *Toxoplasma* tachyzoites differentiate into bradyzoites residing in tissue cysts, which also divide by endodyogeny. Infection of a cat leads to several rounds of merogony in the epithelial cells of the small intestine. At this stage, the parasite divides by endopolygeny, which involves repeated rounds of S phase and nuclear division, followed by the formation of multiple daughters by a final mitosis round linked to internal budding. Subsequently, the parasite sexually differentiates into either microgametocytes, forming multiple biflagellated microgametes, or macrogametocytes, forming a single macrogamete (7). The macrogametes, following fertilization, develop into unsporulated oocysts that are secreted into the cat feces, wherein meiosis proceeds in the extracellular environment, resulting in the formation of eight sporozoites per oocyst. Upon ingestion by an intermediate host, the sporozoites excyst in the intestines, invade through the epithelium of the small intestine, and differentiate into tachyzoites, which completes the life cycle.

**FIG 1:**
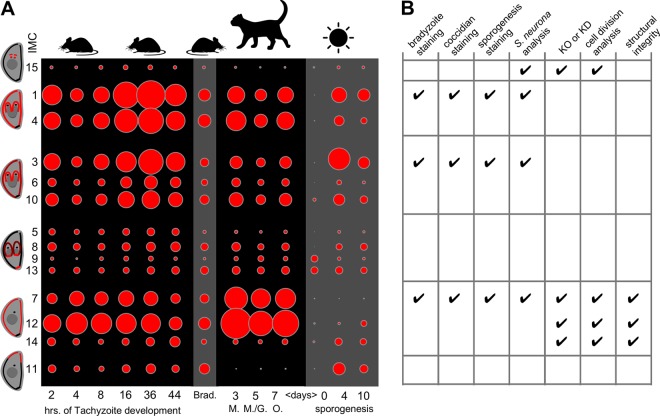
Transcriptomic analysis and experimental overview of oocyst, sporocyst, tachyzoite, and bradyzoite development in *Toxoplasma.* (A) Bubble plot showing the relative mRNA expression levels of all IMC proteins collected by RNA-seq at the stages indicated (days indicate the times of induction and/or growth of the stages indicated). IMC proteins that belong to the same functional group as defined are grouped by their spatiotemporal localization pattern in tachyzoites as shown on the left (10). Bubble size matches the relative level of expression representing transcript levels in FPKM. At the top, the places where the particular development takes place are indicated by graphics, where the mouse represents any intermediate host, the cat any feline, and the sun the extracellular environment. Brad, bradyzoites; O, oocysts; M, merogony; G, gametogony. (B) Overview of the experiments reported in this paper. KO, knockout; KD, knockdown.

Cell division of the nonsexual stages is driven by assembly of the cortical cytoskeleton composed of flattened alveolar sacs undergirded by an intermediate filament network and microtubules (8). This cortical membrane complex is called the inner membrane complex (IMC). The intermediate filament-like proteins are known as IMC proteins containing an alveolin repeat composed of valine- and proline-rich regions (9). *Toxoplasma* encodes a family of 14 alveolin domain-containing IMC proteins that are sequentially assembled into the cytoskeleton of budding daughter tachyzoites and mark distinct developmental steps in the budding process (10). Assembly of the IMC filament skeleton in *Toxoplasma* starts with recruitment of IMC15 to the duplicated centrosomes, from which IMC15 transitions to the first outlines of the daughter cytoskeletons (10) (Fig. 1A, left margin). Subsequently, a large group of IMC proteins, IMC1, -3, -4, -5, -6, -8, -9, -10, and -13, is recruited to the forming daughter buds. However, the behavior of these proteins resolves into several groups. IMC1 and -4 remain associated with the cytoskeleton upon maturation, whereas IMC3, -6, and -10 are partially released upon maturation. IMC5, -8, -9, and -13 transition to the basal end of the budding cytoskeleton when the basal ends start contracting to taper the forming daughters. Although the dynamics of IMC11 are not well understood, it localizes to the apical cap in mature parasites. Finally, IMC7, -12, and -14 associate only with the mature cytoskeleton and are never present on the forming daughters. Here we set out to understand the details of these different groups of IMC proteins; the experiments performed are summarized in Fig. 1B.

Although *Toxoplasma* divides by internal budding, most apicomplexan parasites divide by schizogony. Schizogony unfolds through the formation of multinucleate (MN) schizonts (multiple rounds of S phase each followed by karyokinesis) in a shared cytoplasm and concludes by the formation of daughter parasites at the periphery of the cell, rather than by budding internally as observed in *Toxoplasma* or *Sarcocystis*. The alveolin IMC family of *Plasmodium berghei*, which harbors only seven IMC paralogs, has been studied throughout its life cycle (11–14). In contrast to the *Toxoplasma* tachyzoite, *P. berghei*, which asexually divides by schizogony, expresses only a limited set of IMC proteins per life stage. For example, PbIMC1c is incorporated into the cytoskeleton upon completion of division (12) (comparable to *Toxoplasma* IMC7, -12, and -14), PbIMC1a is expressed in sporozoites, and PbIMC1b is expressed in ookinetes (15), whereas PbIMC1c and PbIMC1e are expressed differentially in gametocytes (13). These different dynamics likely reflect distinct functions across the development stages, as the *Plasmodium* IMC1 family proteins have been demonstrated to be critical for cell morphology, tensile strength, motility, and infectivity (12, 15–18). We therefore hypothesize similar functions for at least some *Toxoplasma* proteins, though additional functions in *Toxoplasma* can be imagined. For example, IMC15 is likely involved in daughter budding initiation, whereas IMC5, -8, -9, and -13 likely contribute to basal complex constriction. Furthermore, *Toxoplasma* must differentially stabilize the mother cytoskeleton (which disassembles late in division) from the budding daughters since budding is internal (unlike in *Plasmodium* schizogony, which forms new daughters in the absence of a mother IMC). Thus, it can be imagined that IMC proteins such as IMC7, -12, and -14 differentially mark mother and daughter cytoskeletons to control the stability of mother and daughter cytoskeletons.

In this study, we show that IMC1 and -3 associate with daughter cytoskeletons, whereas IMC7 is specific for the mother cytoskeleton across the various asexual division modes. Heterologously expressed IMC15 associates with the very early *Sarcocystis* daughters, and IMC3 is enriched in budding daughters. However, unlike in *Toxoplasma*, IMC7 associates with the apical end of *Sarcocystis* daughters rather than with the mother’s cytoskeleton. IMC7, -12, and -14 are not essential for the tachyzoite lytic cycle. We found evidence of slightly varying functions for IMC7, -12, and -14 in the structural integrity of extracellular parasites. Moreover, we found that both *Toxoplasma* IMC14 and IMC15 are required to consistently limit the number of offspring per division round to two, suggesting a putative mechanism to switch between endodyogeny and endopolygeny. Overall, we conclude that different IMC proteins have specific and distinct roles in different developmental stages, which appear to diverge between different parasite species.

## RESULTS

### *Toxoplasma* alveolin IMC protein family expression throughout the life cycle.

To obtain indications of whether particular IMC proteins might be stage-specifically expressed, as reported for *P. berghei* (12), we first mined the gene expression data available in the *Toxoplasma* genome database (http://toxodb.org/toxo/) (19). Transcriptome sequencing (RNA-seq) data are available for tachyzoite development, bradyzoites, the sexual development stages in the cat gut, unsporulated oocysts, and during sporulation (Fig. 1A). The RNA-seq data through tachyzoite development follow the same general profile as observed by Affymetrix microarray analysis (10, 20), but with an expanded dynamic range. They also confirm that groups of IMC proteins with the same spatiotemporal dynamics have matching expression profiles. The profile observed in bradyzoites does reflect the profile in tachyzoites. This suggests that IMC proteins will behave the same in tachyzoites and bradyzoites, which matches with the similar endodyogeny division strategy followed by these two stages (21).

Expression data on the diverse (pre)sexual stages developing in the cat gut were obtained from a time course of infection. Although the general pattern is again quite comparable to the tachyzoite and bradyzoite data, the IMC7 and IMC12 expression level is much higher, whereas the IMC11 expression level is much lower. Throughout the different time points, not much variation is observed, likely because the stages are mixed to some extent because of the low synchrony in the coccidian development of *T. gondii*. This suggests prominent roles for IMC7 and -12 during merogony, when the parasites divide by endopolygeny, forming 8 to 16 daughters per division round (the *Toxoplasma* endopolygeny mode differs from *Sarcocystis* endopolygeny in that each S phase is followed by mitosis), and/or during macrogametogony, where the mother cell IMC is retained (22).

In the oocyst and during sporogenesis, the profiles are much different in that most IMC protein transcript levels are low, consistent with the absence of an IMC in the unsporulated oocyst (22). However, IMC3 and -11 appear to be relatively highly expressed, suggesting specific roles in the oocysts, potentially related to sporoblast formation. Notably low are IMC7 and -12, suggesting marginal roles during this process, which fits the absence of an IMC structure in both the unsporulated oocyst and sporoblasts as new IMC protein only appears upon sporozoite formation (22). In addition, the data show that the groups of IMC proteins based on observations in tachyzoite development do not share expression profiles during sporogenesis, suggesting differential roles for IMC proteins within these groups. Overall, the RNA-seq data match the functional insights available and at the same time predict divergent functions where experimental insights are sparse.

### *Toxoplasma* IMC protein dynamics in bradyzoites.

**FIG 2:**
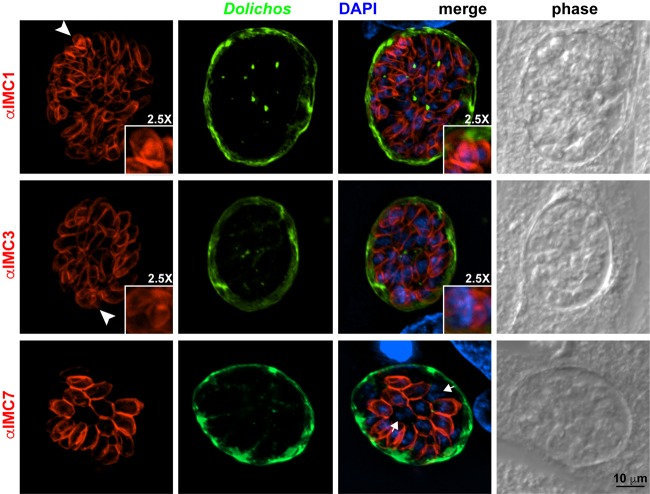
IMC proteins in bradyzoites. IFA of bradyzoites with IMC1, IMC3, and IMC7 antisera counterstained with *D. biflorus* agglutinin lectin to highlight the tissue cyst wall surrounding the bradyzoites and DAPI to label the nuclear material. Arrowheads mark dividing parasites (the bottom right inserts are 2.5-fold magnifications of the marked daughter buds), and arrows point to two parasite nuclei not surrounded by an IMC7 signal.

To verify the mRNA expression data in the different life stages, we chose representatives from three different groups of IMC proteins with putatively distinct roles in different life stages. IMC1 and IMC3 are both associated with daughter buds in tachyzoites, but IMC3 is partially released from the cytoskeleton upon maturation into mature parasites. IMC7 was selected as it only associates with the mature cytoskeleton in tachyzoites. We used a mouse monoclonal antibody (MAb) against IMC1 and polyclonal rat antisera against IMC3 (10) and IMC7, which is first reported here. We performed *in vitro* differentiation to assess IMC protein dynamics in bradyzoites. Bradyzoite formation was validated by staining with *Dolichos biflorus* agglutinin, a lectin that is specific for the tissue cyst wall. We detected IMC1, -3, and -7 in the mature bradyzoite cytoskeletons. We also observed IMC1 and IMC3 in dividing parasites (Fig. 2, arrowheads). Some parasites do not stain with IMC7, suggesting that these have recently completed cell division as the addition of IMC7 to the mature IMC lags slightly behind the completion of division (Fig. 2, arrows). These data confirm recent observations for IMC3 in bradyzoites (21). Altogether, these observations fit with the mRNA expression data (Fig. 1) and show that the (spatio)temporal localization of these three representative IMC proteins is the same in tachyzoites and bradyzoites.

### *Toxoplasma* IMC protein dynamics during merogony.

**FIG 3:**
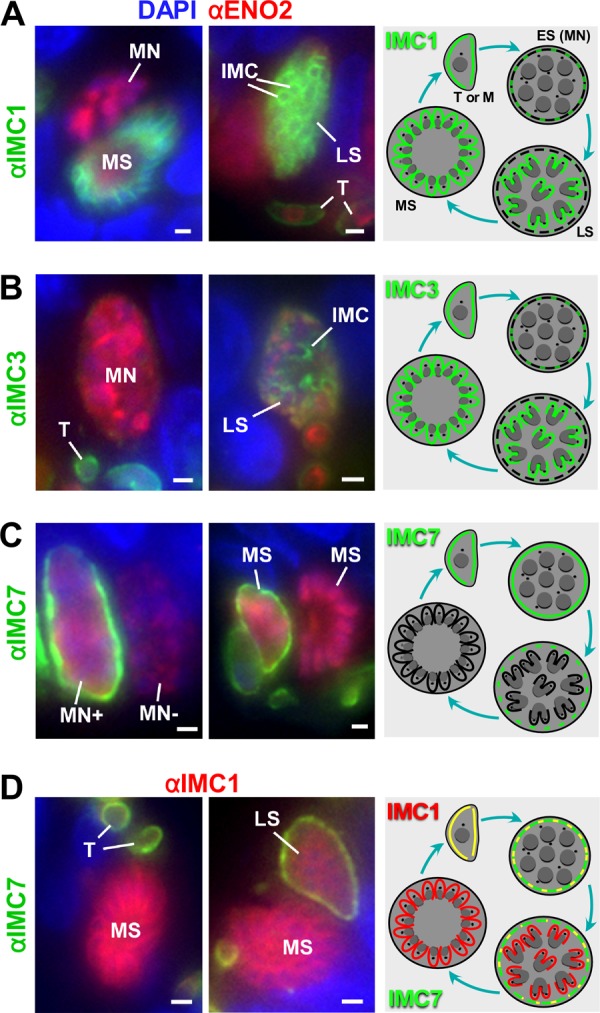
IMC proteins in merozoites and during schizogony. IFA with IMC1 (A), IMC3 (B), IMC7 (C) and costaining with IMC1 and IMC7 (D). Anti-ENO2 costaining was used to highlight the parasites (A to C). The marked stages in both the IFA and schematic are multinucleate (MN; this can be either an early schizont [ES] or early microgametocyte), LS, and MS. T and M mark trophozoites (early growth phase of schizont) and merozoites, respectively. IMC7 specifically stains the polyploid mother, whereas IMC1 and -3 are specific to (developing) merozoites. See Fig. S1 to S3 for single-color panels and additional images. Black lines in the schematics indicate the localization and presence of the IMC, whereas the colors represent the IMC proteins considered. Dotted lines indicate fragmented IMC and/or weak IMC protein localization. Red and green overlay is represented in yellow.

Upon infection of cats, invading bradyzoites undergo merogony to produce 8 to 16 merozoites through endopolygenic reproduction. Although the cortex of newly invading tachyzoites and early intracellular developmental stages stains positively for IMC1, -3, and -7, expansion of the polyploid schizont results in a sharply reduced IMC1 signal and complete loss of the IMC3 signal (Fig. 3A, B, and D; see Fig. S1 to S3 in the supplemental material). While macrogametocytes and certain multinucleated (MN) stages retain IMC7 surface staining, it is difficult to morphologically differentiate between MN cells representing early microgametocytes and schizonts. However, it was possible to identify adjacent MN parasites that are either IMC7 positive or negative (MN^+^ and MN^−^ in Fig. 3C). Since microgametes have been shown to lose the IMC during development (23), we may differentiate between early schizonts (IMC7 positive) and microgametocytes (IMC7 negative). In the later stages of schizogony (late schizonts [LS]), it was possible to identify daughter IMC stained with IMC1 (Fig. 3A and D) and -3 (Fig. 3B), while IMC7 (Fig. 3C and D) staining was limited to the mother cell periphery. Mature schizonts (MS), with fully formed merozoites, were strongly stained for IMC1 and -3 but completely negative for IMC7 (Fig. 3D).

10.1128/mSphere.00409-17.1 FIG S1  IMC1 in *T. gondii* gametocytes. IMC1 is green, ENO2 costaining (red) was used to highlight the parasites, and DAPI is blue. Late schizonts (LS); mature schizonts (MS); multiple nucleated cells, which are either early microgametocytes or schizonts (MN); macrogametocytes (Ma); microgametocytes (Mi); and nuclei (n) are marked. Scale bars, 1 μm. Download FIG S1, TIF file, 2.5 MB. Copyright © 2017 Dubey et al. 2017 Dubey et al. This content is distributed under the terms of the Creative Commons Attribution 4.0 International license.

10.1128/mSphere.00409-17.2 FIG S2  IMC3 in *T. gondii* gametocytes. IMC1 is green, ENO2 costaining (red) was used to highlight the parasites, and DAPI is blue. Late schizont (LS); mature schizonts (MS); multiple nucleated cells, which are either early microgametocytes or schizonts (MN); macrogametocytes (Ma); microgametocytes (Mi); and nuclei (n) are marked. Scale bars, 1 μm. Download FIG S2, TIF file, 2.3 MB. Copyright © 2017 Dubey et al. 2017 Dubey et al. This content is distributed under the terms of the Creative Commons Attribution 4.0 International license.

10.1128/mSphere.00409-17.3 FIG S3  IMC7 in *T. gondii* gametocytes. IMC1 is green, ENO2 costaining (red) was used to highlight the parasites, and DAPI is blue. Late schizont (LS); mature schizonts (MS); multiple nucleated cells, which are either early microgametocytes or schizonts (MN); macrogametocytes (Ma); microgametocytes (Mi); and nuclei (n) are marked. Scale bars, 1 μm. Download FIG S3, TIF file, 2.5 MB. Copyright © 2017 Dubey et al. 2017 Dubey et al. This content is distributed under the terms of the Creative Commons Attribution 4.0 International license.

### *Toxoplasma* IMC protein dynamics during sexual development.

**FIG 4:**
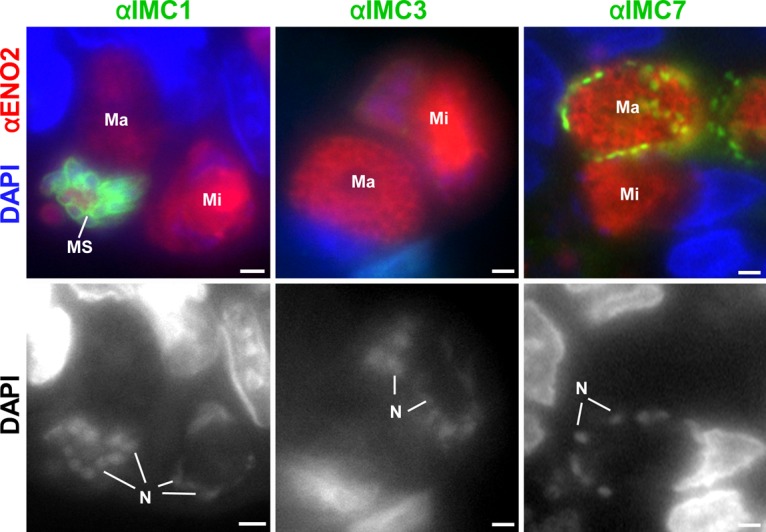
IMC proteins in gametocytes. IFA of *Toxoplasma* gametocyte stages where mature schizonts (MS), macrogametocytes (Ma), and microgametocytes (Mi) are marked. IMC1 and IMC3 were not detected in either gametocyte, whereas IMC7 stained the periphery of the macrogametocyte. See Fig. S1 to S3 for single-color panels and additional images. Scale bars, 1 μm. N, nuclei.

During the progression of sexual development, developing microgametocytes can be differentiated from macrogametocytes because of repeated rounds of nuclear division resulting in ~8 to 16 nuclei while macrogametocytes retain a single nucleus that is diffuse. Developing microgametocytes become negative for IMC1, -3, and -7 (Fig. 4; Fig. S1 to S3). This is consistent with the loss of IMC during microgametogenesis observed by electron microscopy (23). In contrast, during macrogametogenesis, the developing macrogametocyte loses IMC1 and -3 but remains positive for IMC7. In the later stages, there appears to be fragmentation of the cortical IMC7 labeling and some macrogametes were unlabeled for IMC7 (e.g., LMa in Fig. S3). This is consistent with the retention of the IMC during macrogametocyte development and breakdown during the macrogamete-to-oocyst transition with associated wall formation (22).

### *Toxoplasma* IMC proteins during sporogenesis and in sporozoites.

**FIG 5:**
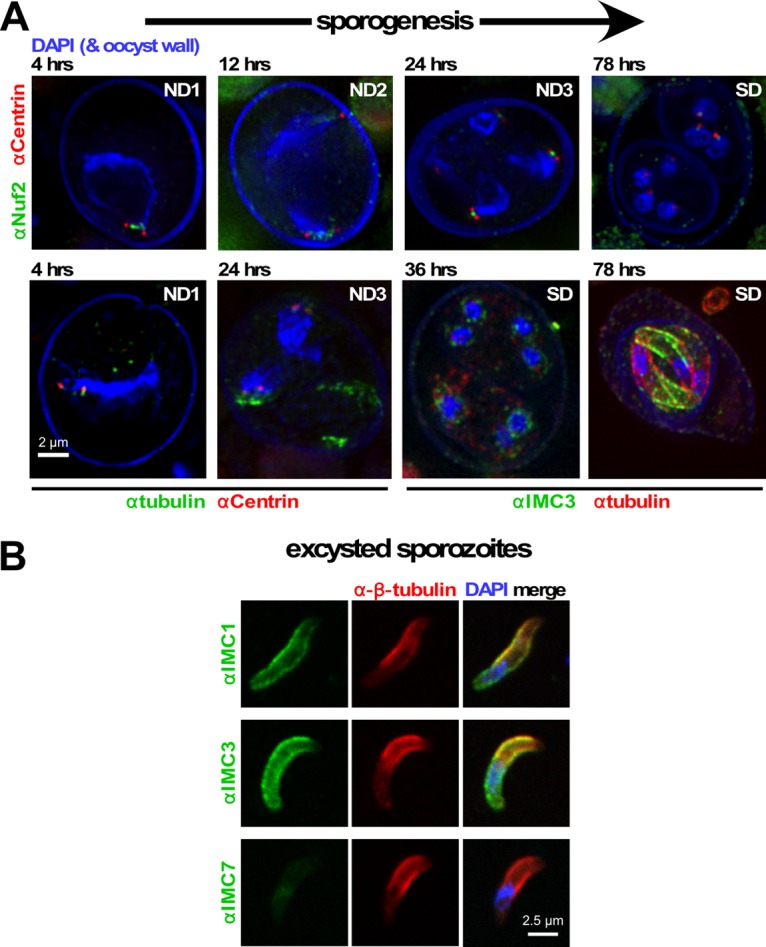
IMC development during sporogenesis and in sporozoites. (A) Sporogenesis tracked markers for the nuclear cycles and sporozoite development. Centrosomes (centrin) and kinetochores (Nuf2) control three nuclear divisions (ND1 to ND3) in sporulating *Toxoplasma*. Anti-β-tubulin staining marks developing sporozoites, while IMC3 shows up relatively late during sporozoite formation. Oocyst and sporocyst walls autofluoresce blue. Nuclei are labeled with DAPI. SD indicates sporozoite development. (B) Confocal laser scanning microscopy of *in vitro* excysted sporozoites stained with IMC1, IMC3, and IMC7 antisera. Parasites were costained with antiserum generated against *Toxoplasma* β-tubulin, which visualizes the subpellicular microtubules and identifies the apical end (top). IMC7 is not present in sporozoites, but IMC1 and -3 are.

Infected cats shed oocysts into the environment, where further development into sporozoites occurs. The RNA-seq data display very low expression levels of all IMC proteins in unsporulated oocysts and only a select set of IMC proteins expressed at relatively modest levels during sporulation (Fig. 1). Consistent with this, we were unable to detect any IMC protein in sporulating oocysts until 36 h after sporulation; defined sporozoites become visible by 64 to 78 h (Fig. 5A). To align sporulation events, in particular the nuclear divisions (ND1 to -3) while going through meiosis and a subsequent mitotic round following formation of two sporoblasts, we stained oocysts at various time points with the kinetochore marker Nuf2 and tracked the ploidy level by staining with a centrosome marker (centrin; Fig. 5A, top row). Four hours into sporulation, oocysts undergo ND1, while ND2 occurs between 12 and 24 h and ND3 occurs between 24 and 36 h. Our data also suggest that the nuclear envelope is strongly remodeled or absent in ND1, as shown by the uncondensed nature of the 4',6-diamidino-2-phenylindole (DAPI)-stained DNA (Fig. 5A). We started to detect IMC3 clusters assembling around the nuclei following the completion of ND3, which subsequently transitions into the sporozoite cortex concurrent with the formation of the subpellicular microtubules (Fig. 5A, bottom row, left two images). Taken together, our data confirm that IMC proteins are not needed for sporoblast formation and indicate that sporozoite assembly might be independent of mitosis, which would set it apart from asexual tachyzoite, bradyzoite, and sporozoite formation.

We note that in order to make unsporulated oocysts permeable to antibodies, the fixation and permeabilization conditions are harsh and may affect the structures within the oocysts, leading to potential artifacts. To partially address this concern, we also tested our IMC antisera on *in vitro* excysted sporozoites so that the fixation and permeabilization conditions were comparable to those used for the asexual stages. Here we observed both IMC1 and IMC3 signals, but IMC7 could not be detected (Fig. 5B). Thus, the indirect fluorescent-antibody assay (IFA) data match the mRNA expression profiles in sporozoites, where IMC7 is poorly expressed (Fig. 1A). Overall, mature sporozoites do share IMC1 and -3, but not IMC7, with tachyzoites, bradyzoites, and merozoites.

### IMC protein dynamics in *S. neurona* endopolygeny.

All asexual stages of *Apicomplexa* within the cyst-forming coccidia (e.g., *Toxoplasma*, *Neurospora*, *Sarcocystis*, and *Frenkelia* but not *Eimeria* species) divide by slight variations on endopolygeny; during *Toxoplasma* endopolygeny, S phase is always followed by mitosis, but during *S. neurona* endopolygeny, S phase is not followed by mitosis, resulting in large polyploid nuclei from which 64 daughters bud (24, 25). In all cases, budding of daughters is linked to a final round of S phase and mitosis. We asked how these slightly different division modes compare to each other in terms of IMC proteins. We used the annotated orthologs SnIMC1 (SN3_01100800), SnIMC3 (SN3_00100240), SnIMC7 (SN3_02200165), and SnIMC15 (SN3_01700570) (http://toxodb.org/toxo/; protein sequence alignments are shown in Fig. S4) (26). The antibody against *T. gondii* IMC1 (TgIMC1) is a MAb against an unknown epitope, which did not display any reactivity against *Sarcocystis* merozoites (data not shown). The IMC3 antiserum was generated against a unique, highly variable N-terminal region in *Toxoplasma*, which displayed effective cross-reactivity in *Sarcocystis*, as we reported before (27) (Fig. S5A). The localization pattern of IMC3 was specific for budding daughters, reflective of its behavior during *Toxoplasma* endodyogeny and endopolygeny. For IMC7, we also observed a specific signal by IFA; however, its localization pattern is distinct from the pattern in *Toxoplasma* in that shortly after invasion, the apical cap of newly invading merozoites is marked (arrowhead in Fig. S5B). Upon growth of the early schizont, the signal becomes weak and diffuse in the cytoplasm of the polyploid cell. Once daughter parasites start budding, IMC7 appears on the apical ends, where it remains in emerging merozoites. The role of IMC7 therefore appears to be different in *S. neurona* and resembles what we observed for IMC11 in *Toxoplasma*, though we cannot exclude the possibility that IMC7 antiserum cross-reacts with another IMC protein. It is of note that *Sarcocystis* does not appear to have an IMC11 ortholog (26).

10.1128/mSphere.00409-17.4 FIG S4  Alignment of IMC1, -3, -7, and -15 proteins of *Toxoplasma* (GT1) and *Sarcocystis* (SN3) generated by Clustal Omega. Percentages of identity and similarity were calculated with MBOSS Needle. Yellow highlighted regions mark the sections of the *Toxoplasma* IMC proteins used for polyclonal antiserum generation. The genome of *S. neurona* contains annotated orthologs for IMC1, -3, -4, -5, -6, -7, -8, -10, -12 (two paralogs), -14, and -15 but none for IMC9, -11, or -13 (http://toxodb.org/toxo/) (26). The orthologs and their percentages of identity and similarity to the selected *Toxoplasma* IMC proteins are as follows: SnIMC1 (ToxoDB accession no. SN3_01100800), 72.1% identity and 83.7% similarity; SnIMC3 (SN3_00100240), 56.0% identity and 66.8% similarity; SnIMC7 (SN3_02200165), 24.5% identity and 39.0% similarity; SnIMC15 (SN3_01700570), 25.5% identity and 34.7% similarity. BLAST searches against the annotated *Sarcocystis* SN3 genome showed high similarity to the annotated genes, with *E* values of 0.0, 0,0, 3e-38, and 4e-87 for IMC1, -3, -7, and -15, respectively. The second best hits displayed much weaker *E* values (5e-37, 7e-18, 9.3, and 5e-17 for IMC1, -3, -7, and -15, respectively). Download FIG S4, PDF file, 0.1 MB. Copyright © 2017 Dubey et al. 2017 Dubey et al. This content is distributed under the terms of the Creative Commons Attribution 4.0 International license.

10.1128/mSphere.00409-17.5 FIG S5  IMC3, -7, and -15 in schizonts and merozoites of *Sarcocystis*. (A) IFA with an anti-TgIMC3 antibody to mark the *Sarcocystis* early (ES), late (LS), and mature (MS) schizont stages. Nuclear material is stained with DAPI. (B) IFA with IMC7 marks the apical end of the merozoites (arrowhead), early schizont (ES), late schizont (LS), and mature schizont (MS). (C) *Sarcocystis* schizonts expressing *Toxoplasma* YFP-IMC15 were fixed and subjected to IFA with an anti-GFP antibody to show the localization of IMC15. The white arrow highlights the alveolar seams in the mother’s IMC, and the yellow arrow marks the very early stages of the assembling daughter merozoites at late stages of endopolygeny. Download FIG S5, TIF file, 2.3 MB. Copyright © 2017 Dubey et al. 2017 Dubey et al. This content is distributed under the terms of the Creative Commons Attribution 4.0 International license.

Exploiting the transfection system that has been developed for *S. neurona* (28), we asked whether *Toxoplasma* reporter plasmids (10) could be used to investigate IMC15 in *Sarcocystis*. We transfected a yellow fluorescent protein (YFP) fusion construct under the control of the constitutive α-tubulin promoter into *S. neurona* merozoites. Interestingly, IMC15-YFP highlighted the alveolar sutures in the mother schizont’s IMC (Fig. S5C). However, upon maturation of the schizont, bright IMC15-YFP signals start appearing inside the schizont, which likely mark the initiation of daughter budding on the basis of their number (64 daughters typically form). Thus, the alveolar suture signal is likely an overexpression artifact, as overexpression effects were also observed in *Toxoplasma* with this plasmid (10). A fortuitous insight from this artifact is that the YFP-IMC15 signal clearly shows the presence of an IMC in the mother. Thus, on the basis of its localization pattern, it appears that IMC15 functions similarly in *Toxoplasma* and *Sarcocystis* in early daughter scaffolding.

### IMC15 is not essential for the lytic cycle, but its loss affects the number of offspring.

**FIG 6:**
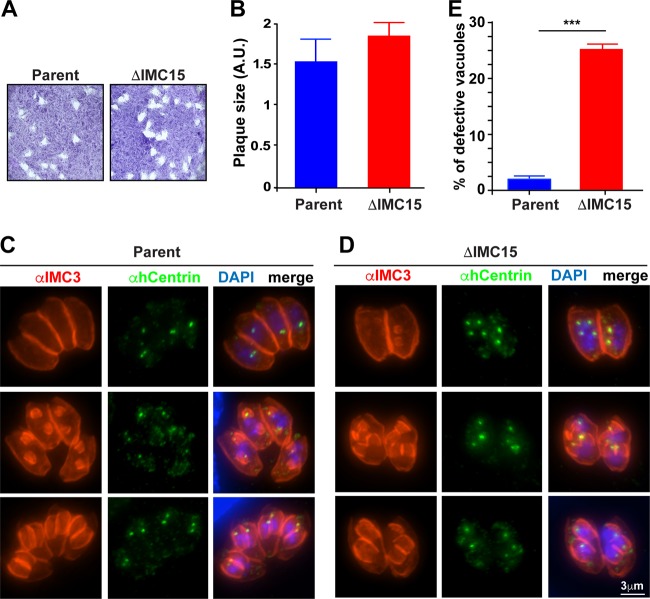
IMC15 is not essential for the lytic cycle, but loss of IMC15 causes a multidaughter phenotype. (A) Representative images of plaque assays of parental (RHΔKu80) and IMC15 dKO lines after a 9-day incubation. (B) Quantification of plaque size (A.U., arbitrary units) of both lines by measurement of the area of 30 plaques. *n =* 3 ± the standard error of the mean. The differences are not statistically significant by unpaired *t* test. (C, D) IFA images of parental (ΔKu80) (C) and IMC15 dKO (D) parasite lines obtained with anti-IMC3 and anti-human centrin (hCentrin) antibodies after methanol fixation. The top row shows the vacuoles with initiating and early daughter buds, the middle row has budding daughters in midphase, and in the bottom row, the daughters are in the late phase of maturation. (E) Graph showing quantification of the multidaughter phenotype by counting 200 vacuoles in each experiment. *n =* 3 + the standard error of the mean. Statistical significance was calculated by using an unpaired two-tailed *t* test (***, *P* < 0.001).

We previously showed that IMC15 is the first IMC protein to be incorporated into the new daughter cytoskeleton (8, 10). Since IMC proteins form oligomers through their alveolin domain, we hypothesized that IMC15’s recruitment to the centrosome prior to its deposition in the daughter cytoskeleton could serve as a scaffold for the recruitment of subsequent IMC proteins to the daughter. In this model, IMC15 is predicted to be essential for parasite budding. To directly probe the function of IMC15, we generated parasites without IMC15. We were able to directly knock out the IMC15 locus (IMC15 dKO; Fig. S6), which, as measured by plaque assays, did not result in loss of viability compared to that of wild-type parasites (Fig. 6A and B).

10.1128/mSphere.00409-17.6 FIG S6  Generation and validation of IMC12, IMC14, and IMC15 knockout parasites. (A) General schematic of dKO strategy by double homologous recombination with CRISPR-Cas9-induced double-strand breaks. Cas9 cleaves DNA at the specific sites, as marked by the yellow arrows. The PCR product of DHFR flanked by the red markers highlights the homologous regions of the IMC15 locus replacing the endogenous locus. The locations of diagnostic PCR primer pairs are numbered I to IV. Gray bars mark the exons of the IMC GOI. (B to D) Diagnostic PCRs to confirm IMC15 (B), IMC12 (C), and IMC14 (D) replacement with primer pairs I to IV as depicted in panel A. Pair IV marks the absence of the GOI, indicating that the replaced fragment has not randomly reintegrated elsewhere in the genome. Download FIG S6, TIF file, 0.9 MB. Copyright © 2017 Dubey et al. 2017 Dubey et al. This content is distributed under the terms of the Creative Commons Attribution 4.0 International license.

To evaluate potential effects of the lack of IMC15 on the budding process, we assessed the early budding steps by IFA using a centrosome (centrin) and daughter scaffold (IMC3) marker (29). As a control, we used the wild-type parent line, which, as expected, showed the formation of two daughter buds around the duplicated centrosomes (Fig. 6C). In the IMC15 dKO line, we observed the same events, but in addition, we frequently observed three or four centrosomes in one mother parasite accompanied by a matching number of daughter buds (Fig. 6D). Although multiple (more than two) daughters form at a 1 to 2% frequency in normal parasites (30), quantification of this phenotype in parasites without IMC15 revealed an increased incidence to 25% (Fig. 6E). Thus, the absence of IMC15 gives rise to multiple centrosomes and culminates in more than two offspring per division round. Taken together, our data suggest that IMC15 is critical for the number of centrosomes and/or daughter buds per division round.

### IMC7, -12, and -14 associate dynamically with the IMC throughout the lytic cycle.

Regarding the putative function of the three IMC proteins only associating with the IMC of nondividing *Toxoplasma* tachyzoites, we generated two models (that are not necessarily mutually exclusive). (i) These IMC proteins may serve as a mark to differentiate mother and daughter cytoskeletons, which is important during the final phases of cell division when the mother’s cytoskeleton is being degraded, whereas the daughters’ IMC is retained. (ii) These IMC proteins may be involved in providing strength to the cortical cytoskeleton of the parasite to withstand the stresses in the extracellular environment and/or during host cell invasion and/or during the expansion of the mother’s IMC during the formation of schizonts during endopolygeny (Fig. 3).

**FIG 7:**
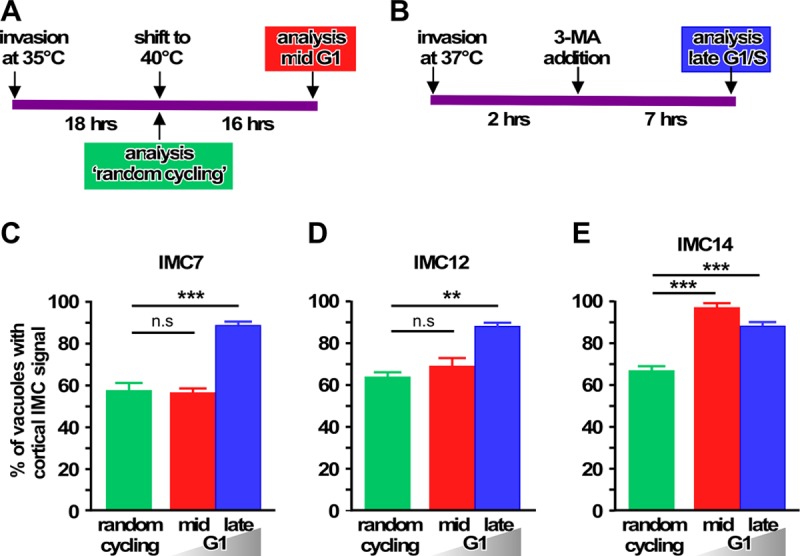
Mature group of IMC proteins completely transitions to the cortex in late G_1_ stage. (A, B) Schematic representations of the procedures used to arrest the parasite in mid G_1_ stage by temperature restriction (A) and in late G_1_ stage upon 3-MA treatment (B). (C to E) Quantification of FV-P6 parasite lines stably expressing mCherry fused to IMC7 (C), IMC12 (D), or IMC14 (E) in the cortex of the parasite after invasion at 35°C (permissive condition; random cycling parasites) and upon mid-G_1_ arrest at 40°C. The percentage of parasites with a cortical IMC7 (C), -12 (D), or -14 (E) signal was quantified after 3-MA block in late G_1_. All graphs were plotted by calculating the percentage of vacuoles with cortical IMC after counting >100 vacuoles. *n =* 3 + the standard deviation. Statistical significance was calculated by using an unpaired two-tailed *t* test. n.s, nonsignificant; **, *P* < 0.001; ***, *P* < 0.0001.

**FIG 8:**
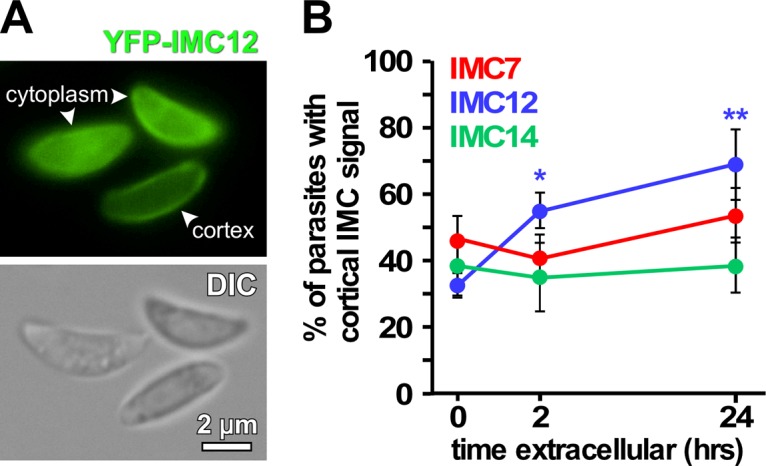
Localization of the mature group of IMC proteins in extracellular tachyzoites. (A) Examples of YFP signal localizing to the cytoplasm and cortex in extracellular parasites stably expressing YFP-IMC12. (B) Quantification of cortical localization of marked IMC signal in extracellular parasites at 0, 2, and 24 h after intracellular release. The graph was plotted from the average percentage of cortical localization of IMC in extracellular parasites determined by counting >200 parasites. *n* = 3 ± the standard deviation. One-way analysis of variance of IMC12 showed significant *P* values between 0 and 2 h (*, *P* < 0.01) and from 0 to 24 h (**, *P* < 0.001), as marked in the graph, suggesting that IMC12 translocates gradually to the IMC, while tachyzoites reside extracellularly. DIC, differential interference contrast.

To differentiate between these two models, we first determined whether these IMC proteins transition in all parasites throughout G_1_ progression, as predicted by model 1. In addition, we wanted to know whether they transition to the cortex in all extracellular parasites, as required for model 2. To test the first model, we assessed the fraction of parasites with a cortical IMC protein upon arrest at two different points in G_1_ by using YFP fusions of these three IMC proteins. We arrested parasites in the middle of G_1_ by exploiting a temperature-sensitive cactin gene mutant named FV-P6, which arrests in mid-G_1_ when grown at 40°C (29) (Fig. 7A; an example of cortical versus cytoplasmic IMC localization is shown in Fig. 8A). In addition, we arrested parasites at the G_1_/S interphase by using 3-methyadenine (3-MA) (Fig. 7B) (31, 32). As a control, we used random-cycling, nonsynchronized parasites (FV-P6 mutant grown at the permissive temperature of 35°C). Our data show that by the end of G_1_, all three IMC proteins transition to the cortex in >90% of all parasites (Fig. 7C to E). This is consistent with the mRNA expression profiles of these IMC proteins, where IMC14 transitions earlier in G_1_ than IMC7 and -12 (10). Critically, the nearly uniform translocation at the end of G_1_ is consistent with a putative role for these IMC proteins in marking the mature mother IMC for degradation.

To test model 2, we mechanically released asynchronous parasites expressing YFP fusions of IMC7, -12, and -14 wherein we scored the fraction of parasites with a cortical YFP signal (Fig. 8A) after performing a time series of extracellular incubations (0, 2, and 24 h). In an asynchronous population at *t* = 0 h, roughly 40% of the parasites display all three IMC proteins in the cortex (Fig. 8B). This percentage only slightly fluctuates for IMC7 and IMC14 over a 24-h period; however, the cortical IMC12 population significantly increases from 32 to 69% in 24 h. These data suggest that IMC7 and -14 are unlikely to have a specific function in the extracellular milieu, but the significant increase in cortical IMC12 suggests that this IMC protein has a potential function in coping with the extracellular environment.

### IMC7, -12, and -14 are not essential for the tachyzoite lytic cycle.

To directly dissect the function of these IMC proteins, we generated parasite lines wherein either the protein level could be conditionally knocked down through replacement of the promoter with a tetracycline-regulatable promoter (IMC7 cKD) or through direct knockout of the locus (IMC12 dKO and IMC14 dKO) (Fig. S6C and D and S7). In the IMC7 cKD line, IMC7 protein becomes undetectable within 24 h of anhydrous tetracycline (ATc) addition (Fig. S7C and D). To evaluate the effect of IMC7, -12, or -14 protein deletion on parasite viability, we performed plaque assays. As shown in Fig. S8, no reduction in either the number or the area of plaques compared to the parental line was observed for any of the mutant lines. These results indicate that when considered individually, IMC7, -12, and -14 are not required to complete the lytic cycle *in vitro*.

10.1128/mSphere.00409-17.7 FIG S7  Generation and verification of IMC7 cKD line. (A) Schematic representation of double homologous recombination to replace the endogenous pimc7 promoter with a drug-selectable marker and a regulatable promoter. Diagnostic primer pairs are numbered 1 to 3. (B) Integration PCRs 1 and 2 to verify the 5′ and 3′ flanks as indicated in panel A in the IMC7 cKD line and the presence of the native promoter in the parental line shown by PCR 3. (C) IFA image showing no IMC7 signal after 24 h of ATc treatment. IMC1 in green marks the cytoskeleton of the parasites, and the DNA material is stained by DAPI. Scale bar, 3 μm (D) Western blot assay to show IMC7 protein downregulation after 24 h of ATc treatment with anti-IMC7 antiserumy. The specificity of the IMC7 antiserum is demonstrated by both the disappearance of the signal in the IMC7 cKD line monitored by IFA and Western blotting, as well as by the size of the band on the Western blot, which closely matches the predicted size of 47 kDa. Anti-α-tubulin MAb 12G10 (αtub) was used as a loading control (bottom). Download FIG S7, TIF file, 2.4 MB. Copyright © 2017 Dubey et al. 2017 Dubey et al. This content is distributed under the terms of the Creative Commons Attribution 4.0 International license.

10.1128/mSphere.00409-17.8 FIG S8  IMC7, IMC12, and IMC14 are not essential for the lytic cycle. (A) Plaque assay of parental (TATiΔKu80) and IMC7 cKD lines after 9 days of growth with or without ATc. (B) Plaque assay of parental (RHΔKu80), IMC12 dKO, and IMC14 dKO lines after 8 days of growth. Download FIG S8, TIF file, 1.4 MB. Copyright © 2017 Dubey et al. 2017 Dubey et al. This content is distributed under the terms of the Creative Commons Attribution 4.0 International license.

### IMC14 restricts the number of offspring per division round.

**FIG 9:**
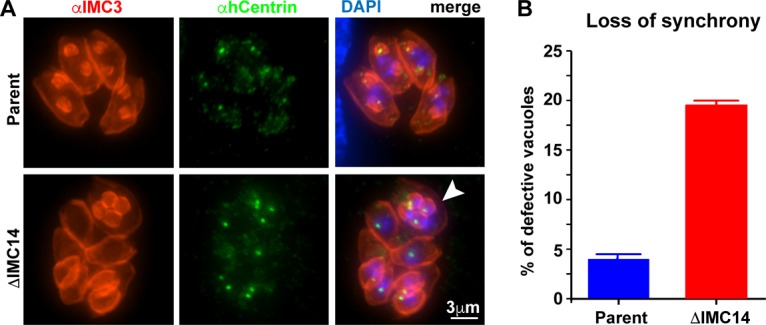
IMC14 is required for synchronous division. (A) IFA images of parental RHΔKu80 and IMC14 dKO lines stained with anti-IMC3 antibody (red), anti-human centrin (hCentrin) antibody (green), and DAPI. The arrowhead indicates a parasite with four budding daughters as opposed to two in other parasites within the same vacuole. (B) Quantification of vacuoles harboring multiple daughters by blindly counting at least 100 vacuoles. *n* = 3 + the standard deviation.

The IMC7-, -12-, and -14-depleted parasite lines provide a new tool to gather additional support for a role for these proteins in either differential mother and daughter IMC function (model 1) or stabilization of the mature cytoskeleton (model 2). We addressed the first hypothesis by performing IFAs with the centrosome and daughter scaffold (IMC3) markers. We observed no discernible aberrations at any stage of the division process for parasites lacking IMC7 or IMC12 (data not shown), but the absence of IMC14 resulted in loss of division synchrony in parasites within the same vacuole, as well as more than two daughters per mother cell (Fig. 9A). This phenotype increased from 3.8% in the wild-type control parasites to nearly 20% of the population in the IMC14 dKO line (Fig. 9B). Overall, the results of these experiments do not support model 1 but instead show that lack of IMC14 surprisingly affects cell division synchrony, as well as the number of daughter buds per division round.

### IMC7, -12, and -14 function in tensile strength.

**FIG 10:**
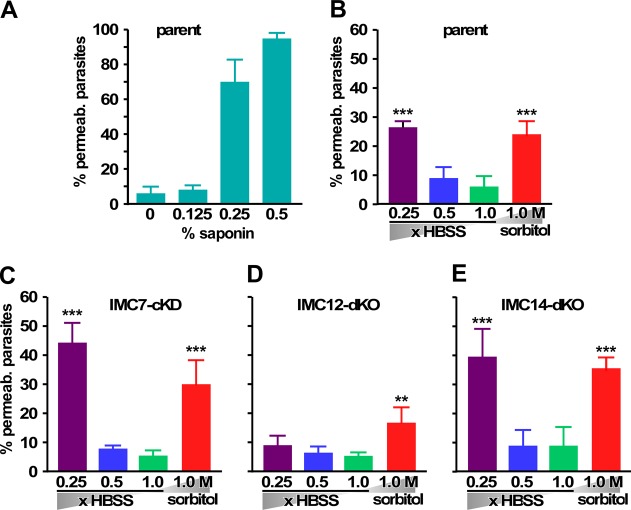
IMC7 and IMC14 are required for osmotic stress resistance. (A) Percentages of permeabilized cells of the control parental line stained with PI after treatment with different concentrations of saponin. Permeabilization was measured by PI staining of the nuclear material by flow cytometry. Alexa 488-conjugated SAG1 was used to differentially stain parasites from debris, which were additionally gated by forward and side light scatter. (B to E) Percentages of permeabilized parasites positive for PI after exposure to hypoosmotic (0.5× or 0.25× HBSS) or hyperosmotic (1 M sorbitol) conditions, as well as a normoosmotic (1.0× HBSS) control. B, RHΔKu80 parental line; C, IMC7 cKD (48 h with ATc); D, IMC12 dKO; E, IMC14 dKO. *n =* 3 + the standard deviation. Two-way analysis of variance compared the hypotonic and hypertonic buffers with control 1× HBSS. *, *P* < 0.01; **, *P* < 0.001; ***, *P* < 0.0001.

To test if IMC7, -12, or -14 is involved in maintenance of the stability of mature parasites (model 2), we asked whether extracellular tachyzoites are able to withstand different osmotic stress conditions (33). The ability to withstand swelling or shrinkage due to water intake or expulsion due to osmotic stress is a general measure of tensile strength. We analyzed parasite integrity through the ability of propidium iodide (PI) to associate with the DNA of ruptured parasites by flow cytometry. We first tested our assay by permeabilizing wild-type parent (ΔKu80) parasites with a dilution series of saponin, which creates pores in the plasma membrane. We observed a direct relationship between the saponin concentration and permeabilization, with nearly 100% of the parasites permeabilized at 0.5% saponin, indicating that the assay works properly (Fig. 10A).

Subsequently, we exposed the parent and IMC-depleted lines to both hypotonic and hypertonic stress conditions by 10 min of exposure to low salt concentrations (Hanks balanced salt solution [HBSS] diluted 2- or 4-fold in water) and 1.0 M sorbitol, respectively. These data show that the permeability of wild-type parasites increases to around 20 to 25% under the most hypo- and hypertonic conditions tested compared to around 7% permeable parasites in standard physiological salt (1.0× HBSS) (Fig. 10B). Although the difference is not statistically significant, IMC7- and IMC14-depleted parasites showed a trend of less resistance to hypotonic conditions than parental parasites (Fig. 10C and E) but withstood hypertonic conditions quite well. Surprisingly, the absence of IMC12 appeared to increase the parasites’ resistance to osmotic stress conditions (Fig. 10D). These results indicate that IMC7, -12, and -14 have distinct and even opposing roles in the maintenance of extracellular parasite rigidity.

## DISCUSSION

Apicomplexan parasites easily adapt their division modes between different hosts and tissues, and we hypothesized that IMC proteins play differential roles in these division modes. Mining of the expression profile data in combination with IFA of representative IMC proteins as tractable markers across different life stages showed that these proteins follow a very dynamic expression pattern unique to each life stage, thereby hinting at stage-specific functions (Fig. 1A). A summary of all of the experiments performed is shown in Fig. 1B. We observed similar localizations of IMC1, IMC3, and IMC7 across the different asexual division modes of *Toxoplasma*. The functions of IMC3 and IMC15 appear to be orthologous in *Sarcocystis* endopolygeny, yet IMC7 displayed a different localization pattern. However, we note that the similarity between the annotated IMC7 proteins in *Toxoplasma* and *Sarcocystis* is relatively low and thus we cannot exclude the possibility that these proteins are not direct (functional) orthologs of each other; in fact, the IMC protein repertoire outside well-conserved IMC1 and IMC3 is quite divergent between these closely related organisms (26) (Fig. S4).

During sexual development and sporulation, the roles of the selected IMC proteins appear to deviate from these rules (Fig. 4 and 5). In the cat gut stages, there is a good correlation with IMC7 staining and the retention and expansion of the mother’s IMC during endopolygeny and macrogamete development. The loss of staining in some mature macrogametes would correlate with the initiation of oocyst wall formation, which occurs while the macrogamete is still within the host cell (22).

The absence of IMC proteins during most of sporulation is consistent with the absence of IMC transcripts in these stages. Furthermore, these observations match electron microscopy observations on the lack of a pellicle structure during development up until the assembly of sporozoites in late sporocysts (34–36). Interestingly, these studies also showed that unlike during endodyogeny and endopolygeny, sporozoite formation is initiated at the plasmalemma (as seen in classical schizogony) (34–36). The uncondensed and irregular, stringy shape of the nuclear material during early sporulation suggests that the nuclear membrane might be absent, or at least strongly remodeled, during these early events. This is consistent with the previous observation that nuclear binding by *Aleuria aurantia* lectin, which preferentially recognizes proteins modified with *O*-fucose and found on *Toxoplasma* nuclear pore complexes and in their proximity, is negative in this life stage (37). Although, electron microscopy also revealed a much enlarged and elongated nucleus, the nuclear membrane could still be identified and appears to be present at all times (36). Collectively, the nucleus and nuclear membrane appear to be altered following fertilization but the nuclear membrane is retained. An additional insight gleaned from our data is that sporozoite budding is independent from a mitotic round, as mitosis and karyokinesis (ND3) appear to be complete before buds start to assemble (Fig. 5). However, electron microscopy data are more consistent with the concurrent occurrence of mitosis with budding (34). The early assembly of IMC3 around the nucleus is very unusual as well. Therefore, we cannot exclude the possibility that other, as yet unknown, cytoskeleton components are recruited before IMC3 or that the harsh fixation techniques used affect the preservation of early daughter structures. However, the formation of the ookinete from the fertilized zygote in *Plasmodium* does occur in the absence of nuclear division and initiates from the plasmalemma (12), so there are examples of mitosis-independent daughter bud formation in members of the phylum *Apicomplexa*.

Thus, when considering the whole life cycle, our data are, in fact, comparable to the observations made in *P. berghei* in that stage-specific patterns emerge. Although the alveolin IMC protein repertoire is twice the size in *Toxoplasma* and *Sarcocystis*, we observed a reduction in the IMC protein repertoire in the sexual stages as reported for *P. berghei* (12). However, a marked difference is that all IMC proteins are expressed in *Toxoplasma* tachyzoites, which is not the case in asexual *P. berghei*. *Toxoplasma* therefore appears to have invested in a much more robust and redundant network of IMC proteins, which is a challenge in the study of these proteins, as redundancy in function is likely.

IMC15, the earliest known cytoskeletal component of daughter cell budding in *Toxoplasma*, is surprisingly not essential for the lytic cycle of the parasite. This observation rejected our hypothesis that IMC15 would recruit additional alveolin domain-containing IMC proteins, which thus appear to be recruited or trafficked independently. However, in the absence of either IMC14 or IMC15, we noticed an increase in multiple daughters per division round, suggesting potential movement into endopolygeny (Fig. 6 and 9). Normally, IMC14 and IMC15 are recruited on the bookends of cytokinesis, so this unified pattern indicates that the switch between endodyogeny and endopolygeny lies on both sides of cytokinesis, in very early G_1_ and at the start of cytokinesis. The centrosome is the structure coordinating these decisions; the inner centrosome controls mitosis, whereas activation of the outer centrosome core triggers daughter formation. Thus, differential activation of inner and outer cores is the basis for endodyogeny versus endopolygeny (38). However, similar multidaughter phenotypes have been observed with other mutations seemingly unrelated to centrosome regulation. For instance, disruption of the GTPase activity of Rab6 shifts cytokinesis toward endopolygeny in 24% of the parasites (39). Rab6 regulates the retrograde transport of proteins between post-Golgi apparatus secretory granules to the parasite’s Golgi apparatus. The deletion of TgNCR1, a protein required for stimulation of lipid storage and membrane biosynthesis, results in an endopolygeny incidence of about 75% because of deregulation of trafficking of the lipids necessary for daughter formation (40). Furthermore, deletion of IMC subcompartment protein ISP2 also leads to a high frequency (60%) of multiple daughters per mother (41). Although the function of ISP2 is unknown, both Rab6 and TgNCR1 are involved in membrane trafficking or biogenesis. It therefore appears that timely availability of membrane is critical for the daughter bud initiation function of the outer centrosome core. Since IMC15 is predicted to be palmitoylated and the IMC meshwork is anchored in the alveolar membranes, a feasible model is that IMC15 assists in recruiting, anchoring, or fusing vesicles trafficking from the Golgi apparatus to start assembly of the new daughter. Thus, if the efficiency of this process is reduced by the deletion of IMC15, it can be envisioned that the outer core will not properly execute its job and subsequently the parasite will forgo daughter development, eventually leading to another mitotic round, producing a total of four centrosomes. The involvement of IMC14, on the other hand, suggests that a decision is made very early in the process as well. This resembles observations in *Plasmodium* where decisions for gametogenesis are made in the schizogony round before differentiation (42). However, it is unclear how IMC14 in the cortex can contribute to this mechanism. Additional data in support of the involvement of IMC14 and IMC15 in the switch from endodyogeny to endopolygeny is the low abundance of their transcripts in both the early and late cat stages.

Regarding IMC7, -12, and -14, which are recruited to the IMC after the completion of cell division, we did not obtain support for a role in the differential control of mother versus daughter cytoskeleton stability in depletion studies, even though all three proteins are present in the mother’s cortex by the onset of S phase (Fig. 7). We cannot, however, formally exclude potential redundancy among these three IMC proteins, which would not manifest in single gene deletions. The alternative function of IMC7, -12, and -14 in the tensile strength of extracellular parasites is supported by the osmotic shock data (Fig. 10). Deletion of either IMC7 or -14 causes a decrease in tensile strength, whereas deletion of IMC12 improves tensile strength. However, these changes are relatively subtle and do not result in decreased viability, implying no or very minor effects on motility or invasion capacity *in vitro* (Fig. S8). Our experiment to monitor the cytoplasm-to-cortex translocation dynamics of the IMC proteins provided some unique insights. We see that the status quo in cortical versus cytoplasmic localization is maintained for the situation at the time of egress for IMC7 and -14. It is well known that parasites are able to complete cell division in the extracellular environment and then end up in G_1_, leading to a G0 arrest (43, 44). Our data now show that parasites emerging during G_1_ largely stay in that stage of G_1_. We interpret this to mean that egressing parasites in which IMC7 and -14 are already translocated to the cortex do not revert back to the middle of G_1_, or at least this does not result in the release of IMC7 and -14. On the other hand, for IMC12, we observe that the association with the cortex slowly increases with the time spent outside a host cell.

Since IMC12 is the only protein whose cortex association increases over time in extracellular parasites, a potential mechanism underlying IMC12 function in extracellular parasites is provided by the observation of phosphorylation of residues S249 and S250 upon egress in a Ca^2+^-dependent fashion (45) (there is no evidence of Ca^2+^-dependent phosphorylation of IMC7 and IMC14). Importantly, phosphorylation events in intermediate filament proteins are a well-recognized mechanism controlling their assembly and rigidity (46, 47). To directly test this model, we generated constructs to genetically complement the IMC12 dKO line with wild-type, as well as phosphomimic (S249E + S250E) and phosphonull (S249A + S250A), alleles flanked at the N terminus by a YFP reporter. Parasite clones stably expressing these alleles were tested for structural integrity (Fig. S9A to C) and cortical translocation efficiency in extracellular parasites (Fig. S9D). We did not observe any significant effects of either mutant allele and therefore conclude that Ca^2+^-dependent phosphorylation of these two IMC12 residues does not provide the mechanistic basis for these two phenomena. An additional mechanism underlying tensile strength was recently reported for PbIMC1c (expressed in tachyzoites, bradyzoites, and sporozoites), i.e., palmitoylation. Palmitoylation anchors PbIMC1c, which is expressed in tachyzoites, bradyzoites, and sporozoites, is a key factor in tensile strength, as it anchors the protein network in the alveolar vesicles (13). Indeed, IMC7, -12, and -14 were all detected in the palmitome of *Toxoplasma* tachyzoites, suggesting that this mechanism could also apply to *Toxoplasma* (48). Thus, it is possible that several parallel mechanisms are at work. Taken together, the results of this study, performed to functionally characterize the members of the IMC protein family, provide evidence of distinct roles for these IMC proteins across different life stages.

10.1128/mSphere.00409-17.9 FIG S9  Ca^2+^-dependent phosphorylation of Ser residues 249 and 250 on IMC12 regulates neither structural integrity (A to C) nor cortical translocation (D). *n =* 3 + the standard deviation. Download FIG S9, TIF file, 0.3 MB. Copyright © 2017 Dubey et al. 2017 Dubey et al. This content is distributed under the terms of the Creative Commons Attribution 4.0 International license.

## MATERIALS AND METHODS

### Parasites.

*T. gondii* RH, RHΔKu80 (49), and TATiΔKu80 (50) parasites and their transgenic derivatives were used in this study. The coccidian stages of *T. gondii* were examined in the small intestine of the cat as described previously (51), the Prugniaud strain was used for bradyzoite experiments, the M3 strain was used for coccidian stages in the cat gut, and the Cz1 strain was used for sporozoite experiments (generous gift from Michael Grigg and Adrian Hehl). All strains were maintained in human foreskin fibroblasts (HFFs) as previously described (52). All parasites were grown at 37°C and 5% CO_2_, except the temperature-sensitive mutant FV-P6, which was cultured at 35°C as the permissive temperature or at 40°C as the restrictive condition. Stable parasite lines expressing transgenes were selected under 20 μM chloramphenicol or 1 μM pyrimethamine based on the resistance marker and cloned by limiting dilution.

A cKD line of IMC7 and dKO lines of IMC12, IMC14, and IMC15 were generated by double homologous recombination under pyrimethamine selection. Parasite lines stably expressing complementing transgenes were selected with chloramphenicol. Gene knockdown in the cKD line was induced with 1.0 μg/ml ATc for times as specified. All of the lines generated were cloned by limiting dilution, and single clones were used for all functional assays.

The coccidian stages of *T. gondii* were examined in the small intestines of cats as described previously (51). All animal experiments were approved by the Institutional Animal Care and Use Committee of Boston University (mouse protocol AN-15306 and cat protocol AN-15294). Oocysts were generated from the type II Cz1 strain of *T. gondii*. For this purpose, 5-week-old female C57BL/6 (Charles River Laboratories, Inc.) were injected in the peritoneum with 10^5^ tachyzoites. During acute toxoplasmosis, mice were administered 95 mg/kg trimethoprim-sulfamethoxazole (Sulfatrim) in their drinking water. Infected mouse brains were isolated 6 weeks postinfection and fed to 11-week-old naive kittens (Liberty Research). Unsporulated oocysts were harvested from cat feces from day 5 to day 10 by sucrose flotation with a specific gravity of 1.15 and subsequently allowed to sporulated in 2% sulfuric acid at room temperature for 7 days as previously described (53). The *in vitro* excystation protocol used for *T. gondii* oocysts was adapted from that used for *Cryptosporidium* (54). In brief, 6 × 10^5^ sporulated oocysts were treated for 30 min with 0.8% (vol/vol) sodium hypochlorite in phosphate-buffered saline (PBS) to remove bacterial contamination. Sporocysts were released mechanically by vortexing for 60 s with 425- to 600-µm glass beads (Sigma). For the release of sporozoites, the liberated sporocysts were incubated at 37°C for 45 min with excystation medium containing 5% (wt/vol) taurocholic acid sodium salt (Sigma) and 0.25% (wt/vol) trypsin (Sigma) in HBSS (Gibco) adjusted to pH 7.8 with 7.5% (wt/vol) sodium bicarbonate (EM Science).

*S. neurona* strain SN3 merozoites and schizonts were used in this study. The parasites were cultured and maintained in bovine turbinate (BT) cell monolayers in T25 flasks. Upon lysis of the infected BT cell monolayer, the merozoites were harvested by passage through 23- and 25-gauge needles and filter purified to remove the host cell debris (55). These extracellular merozoites were used as an inoculum to infect nearly confluent BT cells in 24-well plates containing coverslips. Typically, on day 3 postinfection, the coverslips containing *S. neurona*-infected BT cell monolayers were rinsed gently and methanol fixed for downstream immunofluorescence experiments. Freshly isolated extracellular merozoites were also used for transient expression of YFP-tagged TgIMC proteins in *S. neurona* (28, 56).

### Plasmids.

The sequences of all of the primers used in this study are listed in Table S1. Three-way Gateway cloning (Invitrogen) was employed to generate the IMC7 cKD vector in accordance with previously published protocols (57). Plasmid p*TgKO2-DHFR-T7S4-IMC7* was linearized with ApaI prior to transfection into the TATiΔKu80 strain.

10.1128/mSphere.00409-17.10 TABLE S1  PCR primers and protospacers. All of the protospacers used in this study were phosphorylated at the 5′ end. Restriction enzyme sites are underlined, Att recombination sites are underlined, introns are in lowercase, and mutagenesis sites are in bold font. Download TABLE S1, PDF file, 0.1 MB. Copyright © 2017 Dubey et al. 2017 Dubey et al. This content is distributed under the terms of the Creative Commons Attribution 4.0 International license.

IMC12, IMC14, and IMC15 dKO lines were facilitated by two double-strand breaks at the flanks of the loci induced by the CRISPR-Cas9 system. A 20-bp unique protospacer or guide sequence beside the protospacer-adjacent motif was designed with the program ProtoMatchv2.0 (http://louridolab.wi.mit.edu/) at the 5′ and 3′ ends of the genomic DNA of the gene of interest (GOI). Specifically, sense and antisense protospacers were annealed by denaturation at 98°C for 5 min and slow cooling. The pU6-Sag1 vector (kindly provided by Sebastian Lourido, Whitehead Institute) was digested with BsaI, dephosphorylated, and purified. The purified vector and annealed protospacers were ligated to generate pU6-goi. The plasmid sequence was validated by Sanger sequencing with primer U6_sequence-F. The dihydrofolate reductase (DHFR) minigene was amplified with homologous flanks to the GOI as described in Table S1. Twenty micrograms of each plasmid was transfected into the RHΔKu80 parasite line along with 40 μg of the PCR product of the DHFR resistance marker.

Megaprimer mutagenesis was used to generate point mutations in IMC12 as previously described (24). PCR products were cloned into p*tub-YFP-IMC12/CAT* to generate the YFP-IMC12 phosphomutations with the primers shown in Table S1. Plasmid sequences were validated by Sanger sequencing.

### Transcriptomic analysis.

RNA-seq data, expressed as transcript levels in numbers of fragments per kilobase of exon model per million mapped reads (FPKM), were collected from ToxoDB. The data sets included in the analysis were the tachyzoite transcriptome Time Series (ME49) of Gregory, the bradyzoite *in vivo* transcriptome (M4) of Buchholz et al. (58), transcriptomes of Cat Enteroepithelial Stages (CZ-H3) of Hehl et al. (59), and the Oocyst Time Series (M4) of Fritz et al. (58). Bubbles were generated in Excel, and their sizes correspond to the FPKM numbers.

### Antiserum generation.

Antiserum against IMC7 was produced as described previously (10), with the following specific details. The C terminus of IMC7 (amino acids 228 to 429) was amplified from cDNA and cloned into plasmid AVA0421 to generate a His6 N-terminal fusion. Purified recombinant proteins were used to immunize a rat to generate polyclonal antiserum (Covance). The antiserum was validated for specificity by IFA and Western blotting with the IMC7 cKD parasite line (Fig. S7C and D).

### Plaque assay.

Six-well plates confluent with HFF cells were inoculated with 50 freshly lysed parasites and incubated for 8 to 10 days with or without ATc. Monolayers were fixed with 100% ethanol for 10 min, washed with PBS, and stained with crystal violet (57). Plaques were counted and plaque areas were quantified with Fiji (60).

### Immunofluorescence.

Immunofluorescence assays of tachyzoites, bradyzoites, and sporozoites were performed as previously described (24). Oocysts were resuspended in 4% paraformaldehyde in phosphate buffer and subjected to three freeze-thaw cycles. After at least one overnight incubation with fixative at 4°C, the oocysts were washed in PBS and permeabilized by incubation for 20 min in 0.1% Triton X-100 in 1× PBS (0- to 24-h sporulation time points) or 30 min in 0.25% Triton X-100 in 1× PBS (from the 30-h time point onward). Oocysts were blocked in 3% bovine serum albumin in 1× PBS containing 0.02% NaN_3_. Primary and secondary antibody incubations were performed in solution, and samples were washed by centrifugation for 5 min at 800 × *g*. Images were collected with an Olympus XI70 inverted microscope and deconvolved with DeltaVision SoftWoRx. The primary antibodies used were a rat anti-IMC3 antibody (10) (1:2,000), a rat anti-IMC7 antibody (10) (1:2,000), a guinea pig anti-Nuf2 antibody (57), a rabbit anti-β-tubulin antibody (61) (1:1,000), mouse anti-IMC1 MAb 45:36 (1:1,000; kindly provided by Gary Ward, University of Vermont), a mouse anti-TgENO2 antibody (kindly provided by Stan Tomavo, Université Des Sciences et Technologies de Lille, Lille, France) that weakly stains the cytoplasm and strongly stains the nuclei in actively developing parasites (62), and a rabbit anti-human centrin antibody (1:1,000; kindly provided by Iain Cheeseman, Whitehead Institute). Secondary antisera conjugated with Alexa fluorophores A488 and A594 (Invitrogen) were also used (1:400). Nuclear material was costained with DAPI (2 μg/ml). Images were collected with wide-field microscopes, deconvolved, and adjusted for phase-contrast microscopy with Volocity software (Improvision/PerkinElmer).

### Osmotic shock assay.

Freshly lysed parasites were treated for 10 min with hypotonic buffer (HBSS diluted to 0.5× or 0.25× in deionized water) or hypertonic buffer (1 M sorbitol in HBSS) as previously described (33). Concentrations of 0.125 to 0.5% saponin in HBSS were used as positive controls for permeabilization. Parasites were stained 1:500 with an anti-SAG1 antibody (a kind gift from Jean François Dubremetz, Université de Montpellier) directly conjugated with Alexa 488 for 30 min on ice. Parasites were resuspended in 25 μg/ml PI in HBSS prior to flow cytometry, and data were collected in the phycoerythrin and fluorescein isothiocyanate channels (Becton, Dickinson FACSDiva).

### Western blotting.

Western blotting was performed as previously described (24). A rat anti-IMC7 antibody (1:5,000) (10) and α-tubulin MAb 12G10 (1:2,000; Developmental Studies Hybridoma Bank, University of Iowa) were used as primary antibodies. To reprobe the membrane with a different antibody, it was stripped with stripping buffer (62.5 mM Tris-HCl [pH 6.7], 2% SDS, 100 mM β-mercaptoethanol) for 30 min at 50°C and washed thrice with 1× PBS.

### Bradyzoite induction.

Bradyzoite differentiation was based on a previously described method (63). In short, 3 × 10^5^ freshly lysed parasites were inoculated into the wells of a six-well plate with HFF cells in standard parasite growth medium (Ed1) and allowed to invade the cells for 4 h at 37°C, and then the medium was replaced with Switch medium (1× RPMI supplemented with 100 mM Tricine [pH 8.1] and 1% fetal bovine serum) and the plate was incubated for 4 to 6 days at 37°C in the absence of CO_2_. Immunofluorescence with IMC protein antiserum was performed as described above, whereas the cyst walls were stained with *D. biflorus* agglutinin lectin conjugated with Alexa 594 (Vector Laboratories).
