# Supplementary figures and images for: Differential Roles for Inner Membrane Complex Proteins across Toxoplasma gondii and Sarcocystis neurona Development
Source: mSphere. 2017 Oct 18;2(5):e00409-17. doi: 10.1128/mSphere.00409-17 (PMC5646244; doi:10.1128/mSphere.00409-17)

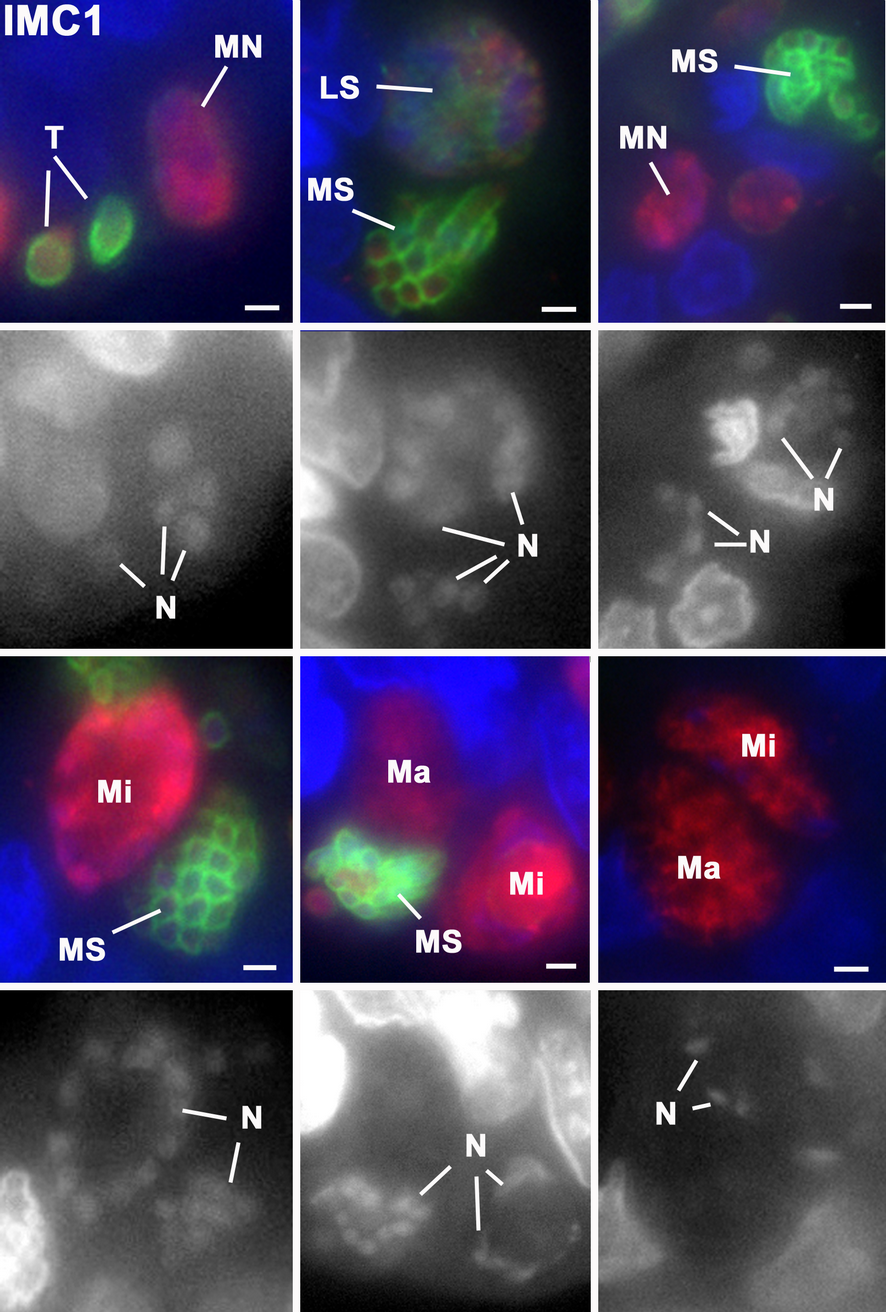

Supplement: FIG S1 [file sph005172388sf1.tif]

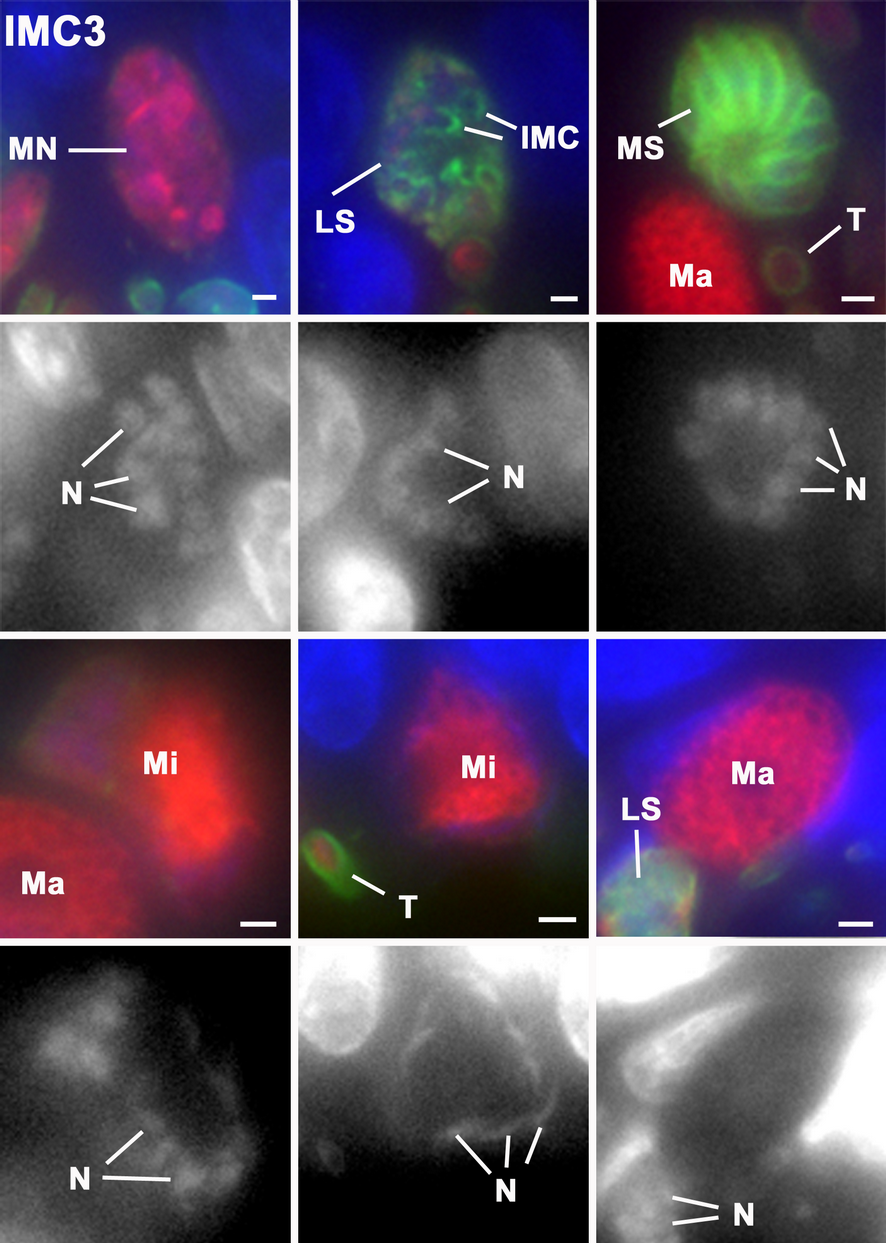

Supplement: FIG S2 [file sph005172388sf2.tif]

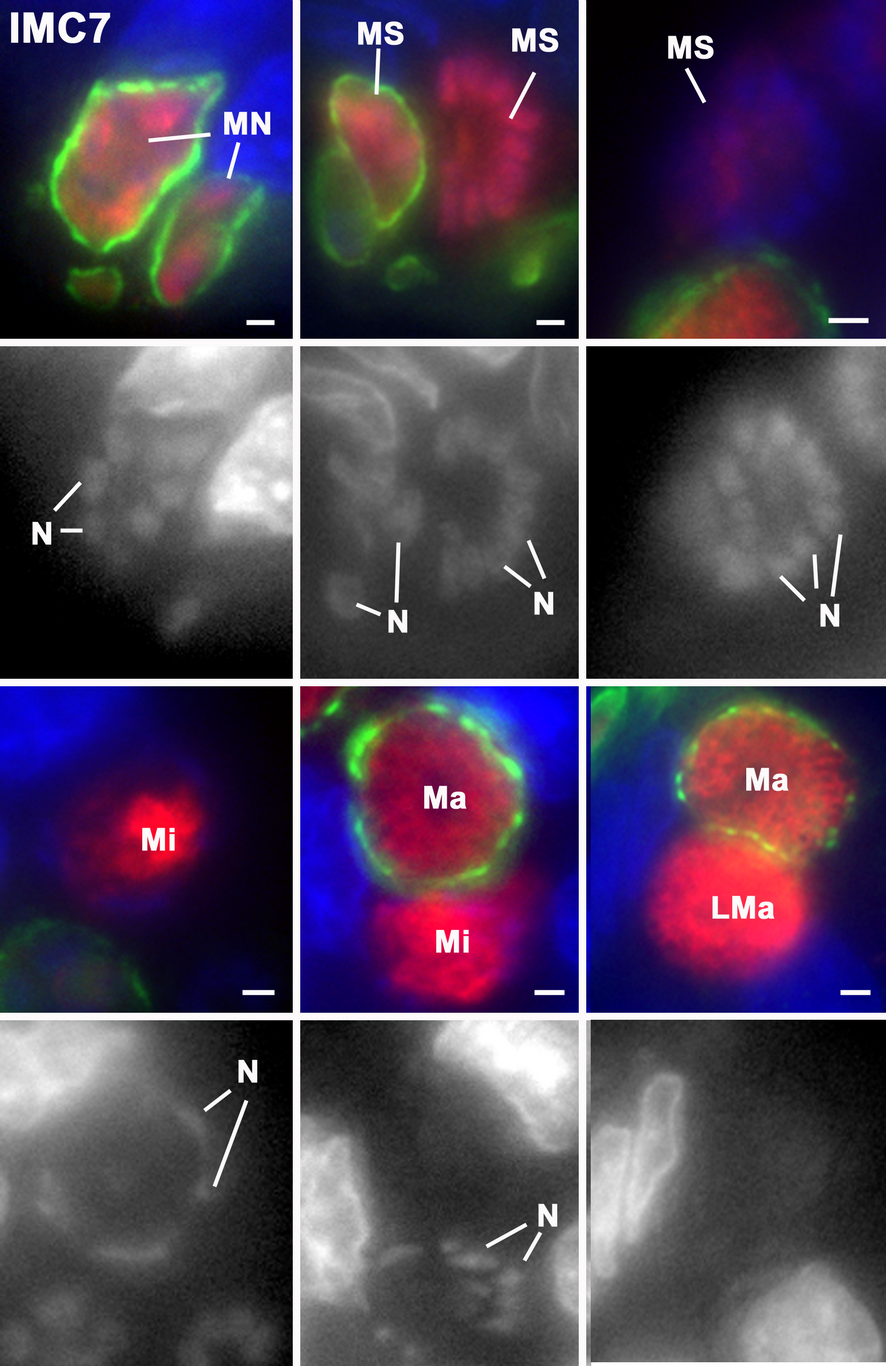

Supplement: FIG S3 [file sph005172388sf3.tif]

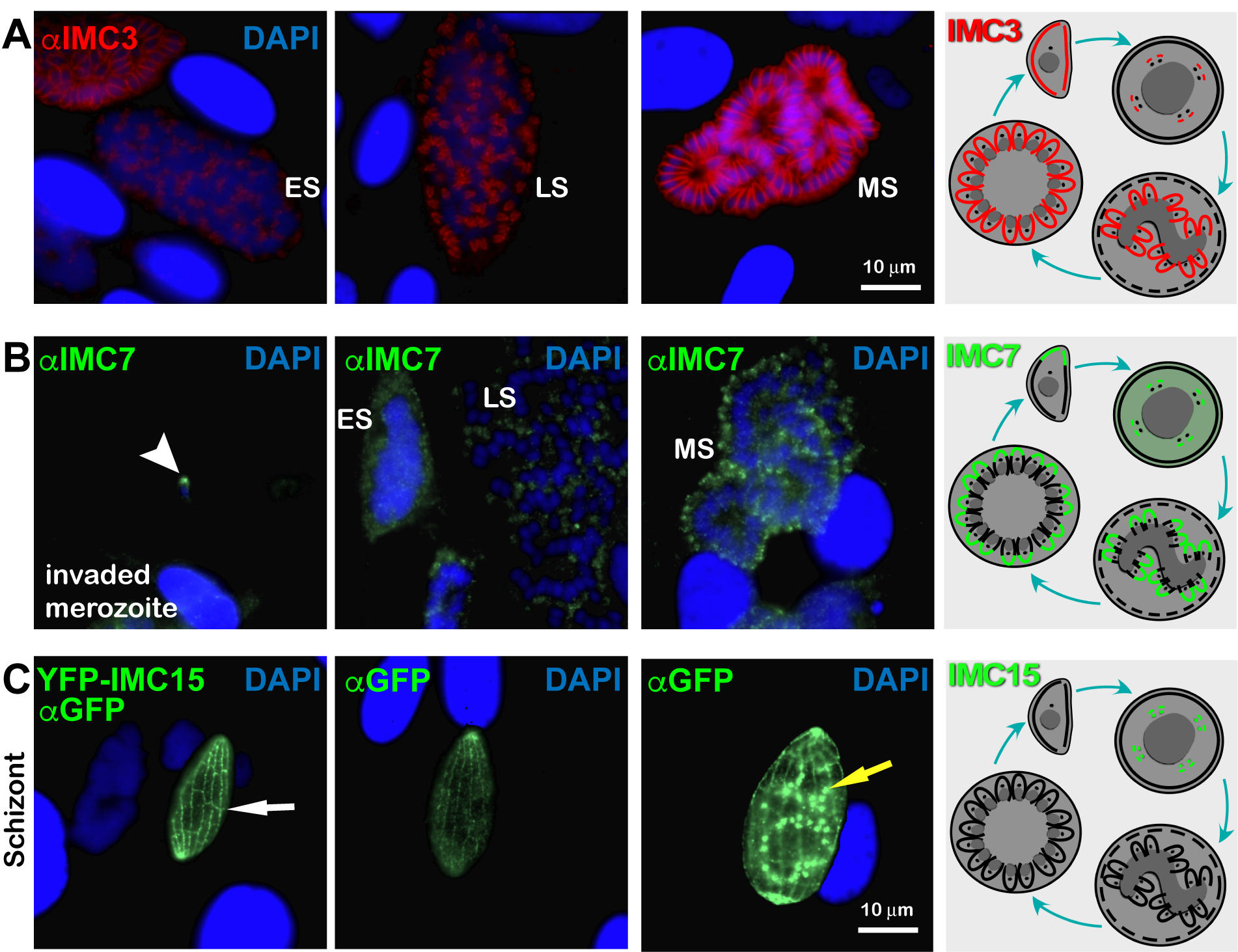

Supplement: FIG S5 [file sph005172388sf5.tif]

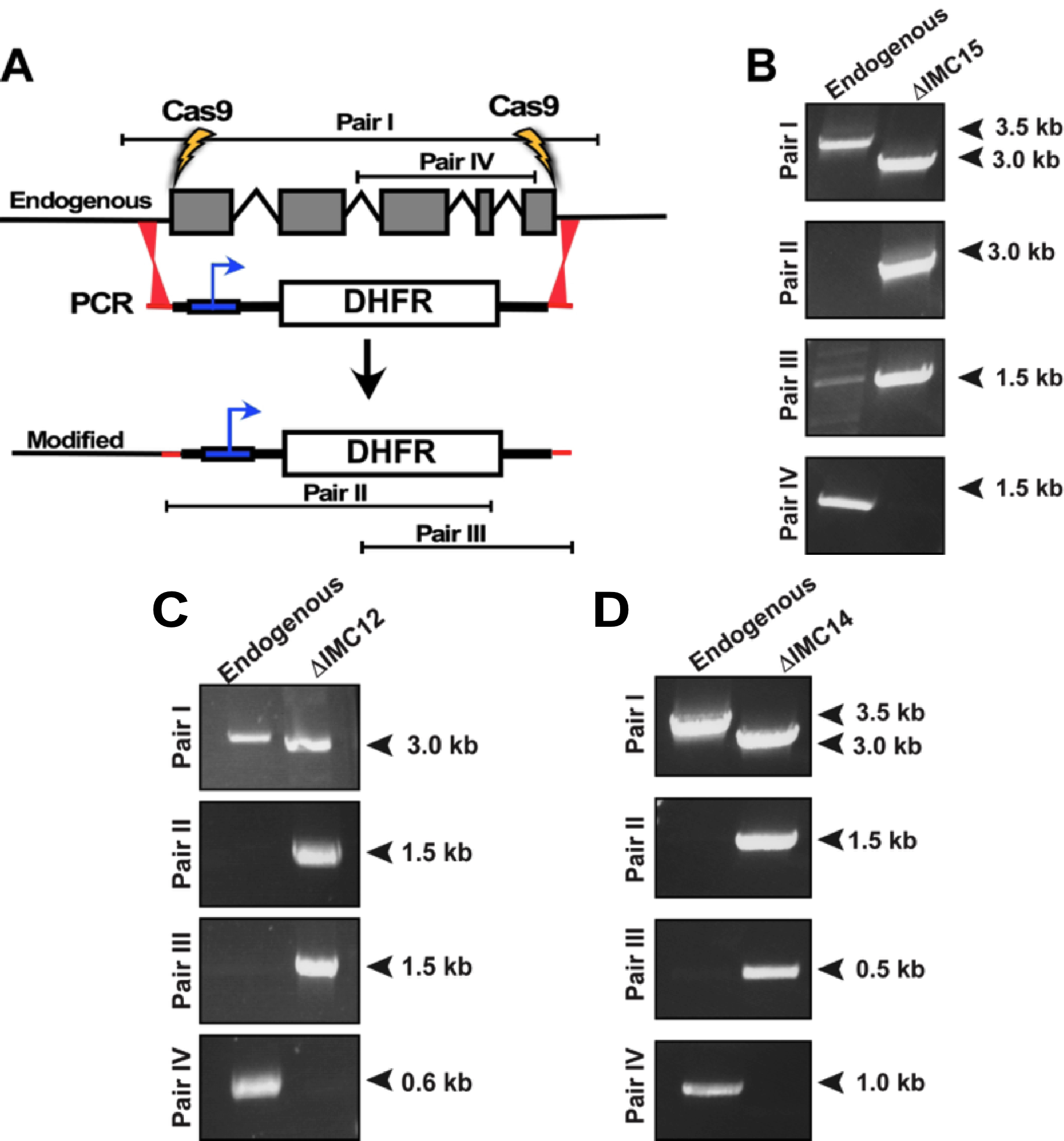

Supplement: FIG S6 [file sph005172388sf6.tif]

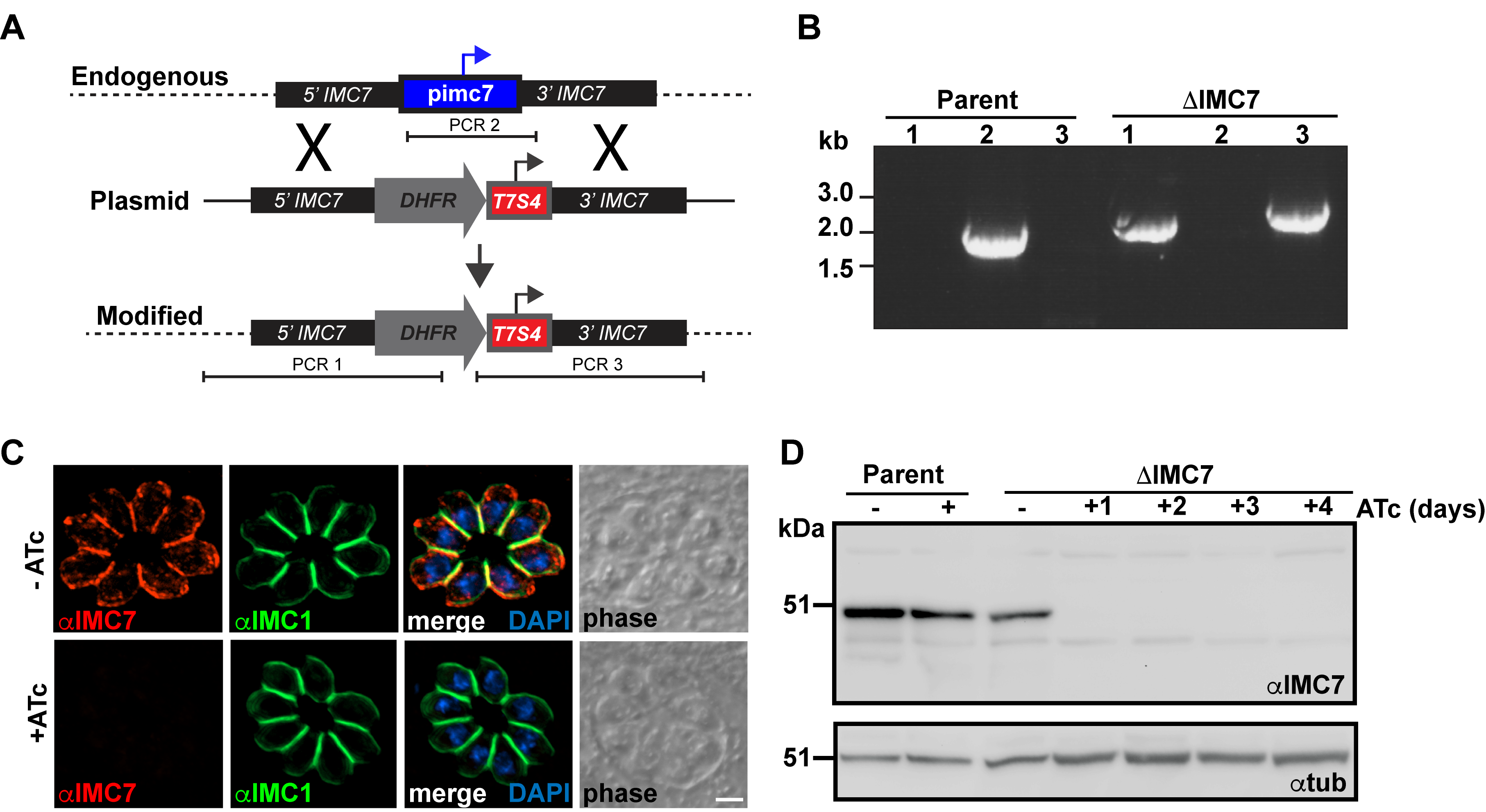

Supplement: FIG S7 [file sph005172388sf7.tif]

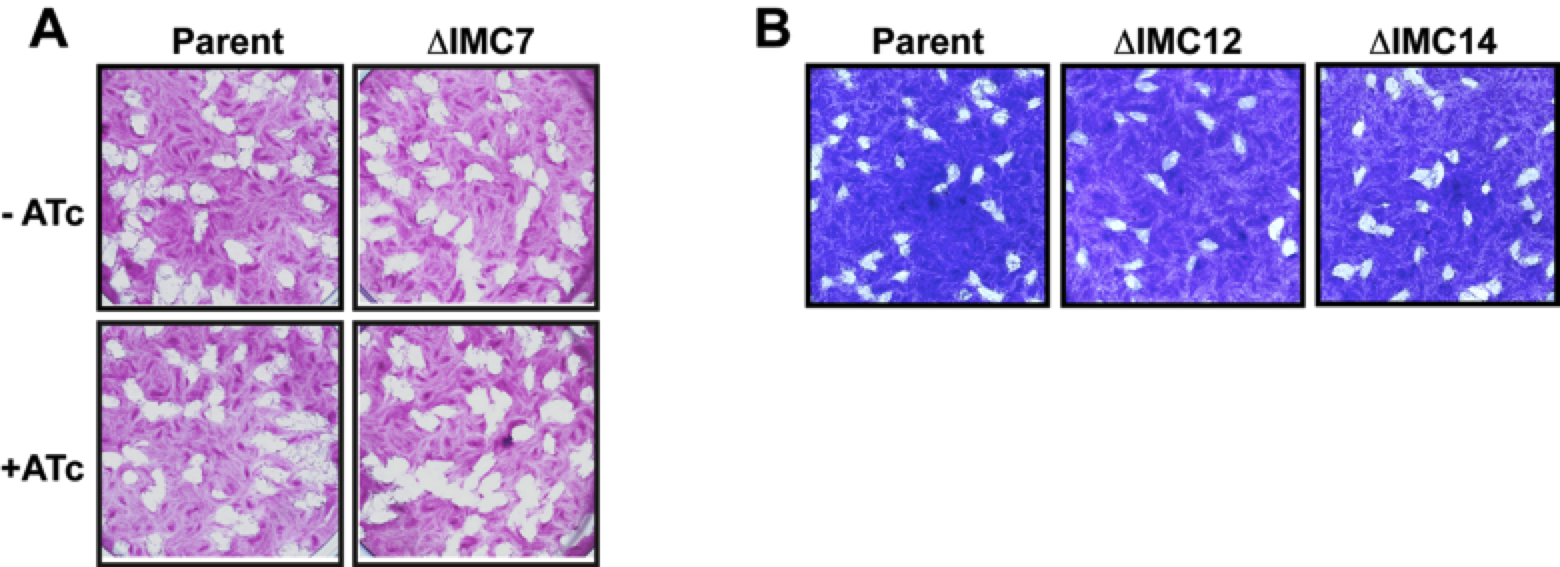

Supplement: FIG S8 [file sph005172388sf8.tif]

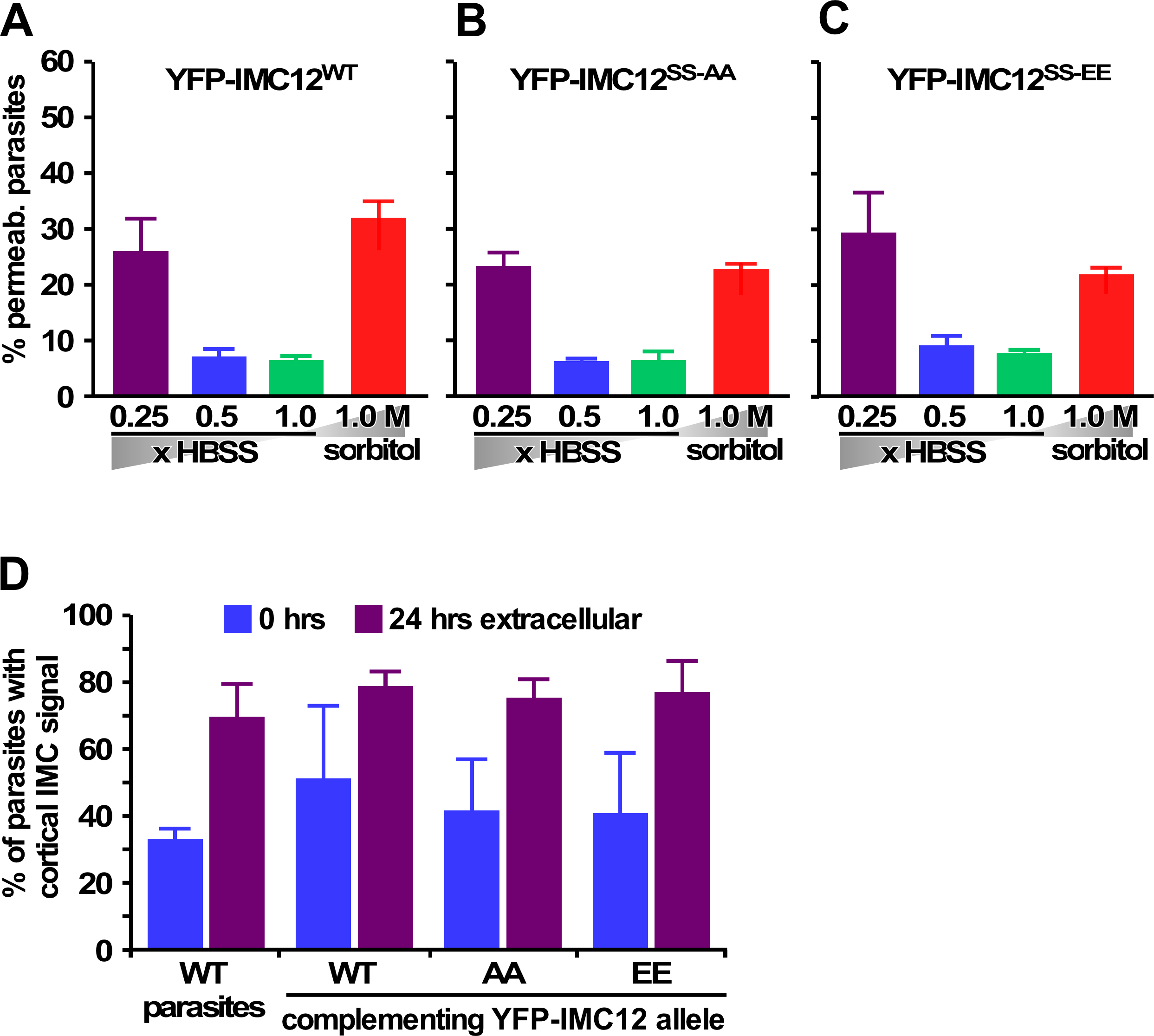

Supplement: FIG S9 [file sph005172388sf9.tif]
