# Supplementary material for: Differential Roles for Inner Membrane Complex Proteins across Toxoplasma gondii and Sarcocystis neurona Development
Source: mSphere. 2017 Oct 18;2(5):e00409-17. doi: 10.1128/mSphere.00409-17 (PMC5646244; doi:10.1128/mSphere.00409-17)
Supplement: FIG S4 [file sph005172388sf4.pdf]

Supplementary Figure S4

IMC1

Identity 72.1%; Similarity: 83.7%

```
TgIMC1      MFKDCADPCSDCCQPAEQQRGQATLPSHVVLPQTSDSPAIHVTAEASQRLS-SPRALQQL    59
SnIMC1      MFQECCEKASNCCQPSDEAALRENVARSSAIGEEGRVAGAA-PAGTSQEQSTNMAESSSS    59
            **::*.: .*:****:::   : .:    .: : .   .    * :**. * .    ..

TgIMC1      HGTQESVRSVTLEERADRSRTVPIGEETERQWVAITAYQPIDTVTKTVEVPVVRTVETYV    119
SnIMC1      TQVQDAYQSVTLEERADRSRTAPVTEQTERQWVAITAYQPIDTVTKTVEVPVVRTVETYV    119
              .*:: :*************.*: *:*********************************

TgIMC1      PKVVVEEKIVEVPKYIPEYVEKVVEVPEVQYVDKIVEVPEYQYEYKYVPKIETKENIIQR    179
SnIMC1      PKVVVEEKIIQVPKYVPEYVEKVVEVPEVKYVDKLVEIPDYQFQYKYVPKVETKENVIQR    179
            *********::****:*************:****:**:*:**::******:*****:***

TgIMC1      PKYETKYIEKVVEVAQVKEVVRYQDVEEVEEIIRYVPKDSVVPEEWKRIQEEQDKREAEL    239
SnIMC1      PKYETKYTEKVVEVSQVKEVVRYQEVEEVEEIIRYVPKGHAIPEEWKRAQEEQQRREEEK    239
            ******* ******:*********:*************. .:****** ****::** *

TgIMC1      KEKEAKEEEELKGLLEEERKLHEERVREQIKVQQQIIQQQLSARAEQERLLKERREAEMV    299
SnIMC1      KEKEAQEKQALEGLLAEEKELQDKRMTDQQRIQQEIVQRQ---RAEHERIIKERKEAERL    296
            *****:*:: *:*** **::*:::*: :* ::**:*:*:*   ***:**::***:*** :

TgIMC1      HFPAVEGAPPLPTIPKVEQVFKPKVIKQVEIQKHVPISVDVPVPYMVPKPVVVSVQVPVL    359
SnIMC1      QYGLPEGAPPMPFIPKVEQVFKPKVVKQVEIQKHVPISVDVPVPYMVPKPVVVPVQVPVL    356
            ::   *****:* ************:*************************** ******

TgIMC1      KFRDHFVPVPVRRRVVPRIRWTNEVYEVECIKEKPFLQVQDVIKPVPCDVEIRVHEFVER    419
SnIMC1      KFRDHFVPVPVRRRVIPRIRWTNEVYEVECIKEKPFLQVQDVIKPVPCDVEIKVHEFVER    416
            ***************:************************************:*******

TgIMC1      AAPINPAELSQADIHAMWMRVNADLAEKRKQELGDKYPYVKHPAGTVFGEPGCSEEEGEV    479
SnIMC1      AAPINPAELSQADIHAMWMRVNADLAEKRKAELGEKYPYVKHPPGTVFGEPGCSEGEGEE    476
            ****************************** ***:******** *********** ***

TgIMC1      ESAQAEEGGQPAGAEPLALHPGHPLNMTYLQNEWIKKSTVTTHEMYTPEWFEAHQKALFN    539
SnIMC1      G-GEAQEGEAREGVEPLALHPGHPLNMAYLQNEWIKKETTATHEMYTPEWFEAHQKALHN    535
              .:*:**    *.*************:*********.*.:*****************.*

TgIMC1      LTMQQPTQVQLSAEQAAKLQQQEAQFGDAPWIEEAHAGVPMKPAAPEPEAAQCCVMCAV-    598
SnIMC1      LTMQHPTQVQLSAEQASQLQQQEPQFGEAPWLDEGVTTQKKGRARDVADSAQCCPVCNSV    595
            ****:***********::***** ***:***::*. :      *    ::**** :*

TgIMC1      -CGGDGVCRCQC     609
SnIMC1      CGGGDGVCKCEC     607
              ******:*:*
```

IMC3

Full length protein identity 56.0%; Similarity: 66.8%

Fragment for antiserum generation identity 33.5%; Similarity: 43.7%

```
TgIMC3      ----------MSDAGT---------------PPAVQGELSQPQERVEVVVQPISESPDAS     35
SnIMC3      MASPEADASSASPAGPGGATDTEVEQTPGIAAERRTGVLTEGGQQVQITIQPIPAL---T     57
                       * **                     * *::  ::*::.:***      :

TgIMC3      SEAEQAGVAAGAFHAEVPYPYPPVSTGMPAPEQPAEMHLPA------------NGQLPEI     83
SnIMC3      GGPGSVQIEERTFHSEVPYPHVPVSATLPSPQEGPKYGGSPAWSAETEAEAGRAVGVPAV     117
            .   .. :   :**:*****: ***: :*:*::  :                    :* :

TgIMC3      -AEGAETEGLAPVARPPPQVMTALGPMPLPPEVRQKIPEKFVAKPIVEEREIYVSKKEVR     142
SnIMC3      YDADEVAGGAGPVARPPPQVMTALGPMPLPAEIRKKIPEKFVAKPIVEEREIFVAKKEVQ     177
               .  : * .******************* *:*:*****************:*:****:

TgIMC3      ERTIEVPHVHYEHKFAEVARSLKIKKLVPRVREVVKEIPREVYKPVIEEKVIEVPQGVKY     202
SnIMC3      ERTVEVPHVHYEHKFAEVATKLKIKKLVPRVTEVIKEVPREVYKPVIEEKVIEVPQGVKY     237
            ***:*************** .********** **:**:**********************

TgIMC3      VEVPVEVPCLYPPKIVPKPKVQVIERIVETIKPVVKEKIVEVPQTVVKQIPKIKTVEVPY     262
SnIMC3      VEVPVEVPCMYPPKIVAKPKIQVVERVVETIKPVVKEKIVEVPQTVVKQIPKIKTVEVPY     297
            *********:****** ***:**:**:*********************************

TgIMC3      YVPRYVEKVIEIPYQPPDAATLPPLMVGGLPSRVSATMPTPFQLGPAPAGHANEVNLPFE     322
SnIMC3      YVPRYVEKVIEIPYQPPDTATLPPMVMGGLPSRITATMPTPFQVGPVPAGQLSEFNLPYE     357
            ******************:*****:::******::********:**.***: .*.***:*

TgIMC3      ARVDVTVSQSPAGPHSPLSASPQTGTAASLK----LGRPATTLVNPDRSAPAIDLPEGFT     378
SnIMC3      AKVDIKVSHGPPTGLSATFPA--PGLLPPQSVGGTDSGAATTRMSPGSPGPAIELPEGFQ     415
            *:**:.**:.*    *    :   *     .     .  *** :.*.  .***:*****

TgIMC3      WGPPPNAPPGTFPA--------------------------------PAMPGLPGAAGLPV     406
SnIMC3      WGPPPNAPPLPTPLCQPVAGSIFSGTPGGGARAREGSVSSLTGLPPSAVPGFPA-SGPAG     474
            *********   *                                  *:**:*. :*

TgIMC3      GAADGLNMPPMMITCPPEVGNVKFAGFLADPDILTRRGVMAYQGFKKEGTFQMLFPPLAP     466
SnIMC3      GGPNVMNMPPMMITCPPEVGNVQFSGYLSDPDILTRSGVMAYQSFKRKGTFQMLFPPLAP     534
            *. : :****************:*:*:*:******* ******.**::************

TgIMC3      QPGIPLDQTGAPDVNTIVSVNEQMAGQSRHHLIEDLQQQVESEFDQK------VNASAMS     520
SnIMC3      QPGVPMNPPGTPDVRTLDQVASQAAGQGRHDVLEAIQKAEEEHERASKAGLASVLSACAP     594
            ***:*::  *:***.*: .* .* ***.**.::* :*:  *..   .      * ::.

TgIMC3      CTPWASPAVQEREVVYEQ         538
SnIMC3      CESCAPEVTPQREIVYEQ         612
            *   *  .. :**:****
```

IMC7

Full length protein identity 24.5%; Similarity: 39.0%

Fragment for antiserum generation identity 23.6%; Similarity: 40.9%

```
TgIMC7      --------------------------------------------MEFT--------ADNA    8
SnIMC7      MKGFTPQAYEGWCQPVAIPIIPNPGEYTMRSGTRWGACCGTWKRMPYQTFPLITTYVRGA    60
                                                        * :         . .*

TgIMC7      APLTEQNAPQDV--EAVKPDVIENAAETANEESAAPTLKRLVSKTTHIKTVTESLSRKQT    66
SnIMC7      LPYIEHEKLMSTNGLAVAPQRAAVKVRVGSGSKTSPMVGNGTQTITMVGSGTQTIPVVGT    120
             *  *::   ..   ** *:     ..... ..::* : . ... * : : *:::    *

TgIMC7      KEI-----TTNTLHMGWKETKEAFLAPRSTITHADGSESEIARFVPSVQVVDLPLDLVYT    121
SnIMC7      QTIPVVGSGTQTIPMVREVTENTCMK-------------------PRVLTKVIECEDTKY    161
            : *      *:*: *  : *::: :                    * * .  :  : .

TgIMC7      VPEVKTRMVNYIFECPARGHSRLVPREFPVDTPFVVPQYQDVSVPVVMSQTFVPELQETS    181
SnIMC7      IPKVEVRTVQRTIEVASKTEKRFVPREVPVDTPFIVPRFEDRDVPVVVSNTISPEIEETE    221
            :*:*:.* *:  :*  :: ..*:****.******:**:::* .****:*:*: **::**.

TgIMC7      RVVQVPVARYVPKLVPVDVFVPRPVAIPIKAGEVTQVSKNTVISDDLMRQLSVEMNPHLE    241
SnIMC7      AVVEVPVARYVPHLVPINVYVPRAVAIPVVLTDESKQEAPAALPTESLQQLIKELNPHLE    281
             **:********:***::*:*** ****:   : :: .  :.:  : ::**  *:*****

TgIMC7      ALNQFNAQQAQVMNNVVARAQELATQMDCPVPSREKIEVNASAGSGAGATIVDEQGNKQL    301
SnIMC7      EIDKFNAGQNVKFQELLEKSQKAANFAKCPVPEPQSTPILKITEIEPEGE-ESDHLVKQI    340
             :::*** *   ::::: ::*: *.  .****. :.  :   :     .   .::  **:

TgIMC7      QLDMGGKELRSVEMVFKRLHDDINTSKCVEENMQITEDIILCVHRDLNGGRLESFPSGNA    361
SnIMC7      CFSTGGYQAAEVESLFTALTATSTTDSEAD---------TTSAHKGMSGEQTDPDT----    387
             :. ** :  .** :*. *    .*.. .:           ..*:.:.* : :

TgIMC7      GIPMIFRAPDQKPSVANGLPERVPLRSLTNDLSHAAAAQNESATTTGEASLPTALNAEEQ    421
SnIMC7      --------HDDMLSAATGASDSQRCTSV---MSGKDATEGVAAVASGKASATTEERKSDA    436
                     *:  *.*.*  :     *:   :*   *::. :*.::*:**  *  . .:

TgIMC7      TTAVPIAA         429
SnIMC7      SKA----A         440
            :.*    *
```

IMC15

Full length protein identity 25.5%; Similarity: 34.7%
Fragment for antiserum generation identity 15.8%; Similarity: 23.8%

```
TgIMC15      ------------------------------------------------------------    0
SnIMC15      MAFDLPKVPRHLPEPGSAAAVPLPSLPFSAAAAATHRPLRCLGTFIVEATPASAGAQTKR    60

TgIMC15      ------------------------------------------------------------    0
SnIMC15      RATRSSSCCHSRCFRSSSCRRYSMTSTSGNRDTSSSSVTASNTANSSQPAGLRDNSKGAS    120

TgIMC15      ----------------------MRICLPPVPQGREAVPDASANVRRQAFSERFRQCEHER    38
SnIMC15      FASLCCNGEAAAAASFQGKYSDVHGCYPPPAAGARGDDGSRNGMPKHVVDQK-----NVA    175
                                   :: * **   * ..  .:  .: ::...::     :

TgIMC15      LPSGVPRPQTPRCSGTILIEPAPTGTNNRQQAPTGRCMDMSGSCASV----------A-E    87
SnIMC15      AAAAVDDCLVPACSRSINGSCFL---RNCCCPEEGAAASVAPSCAPAAAAHVLRPEEAKA    232
               :.*    .* ** :*  .      .*      * . .:: *** .          *

TgIMC15      SAAACCLWQTSPRRWRPTPREPKMCFFEEPSQSL------RFRTPSPRFSCAETTTR--M    139
SnIMC15      GSSCCCGFARRSR-ASSSPSTASPCVPNGLIPRPRVLTAGTAVTPPRRFSFTDLRSATVL    291
             .::.** :    *    :*   . *. :               **  *** ::  :   :

TgIMC15      CTTPCSSTD-----DR-------CTSRRSIYGTPQIQT----------------FSASS-    170
SnIMC15      PSPPARTTAAAPPQEKHQMLQQQLQQQQQLQQQQQLQQQQQQLQQQQQLQQQQQLQQQQQ    351
              : *. :*      ::          .::.:    *:*                 :. ..

TgIMC15      -AGTQAYIRPLGQSSRSVSPWQSALAPASSAVSSTETTH---GIGI-------------Y    213
SnIMC15      QLQQQQQLQQQQQRPFTTFWWEPSTDSASSCCRAEGQAKEQRQLLLHAATAPLPPLSSSL    411
                 *  ::   *   :.  *: :   ***.  :   ::    : :

TgIMC15      ATQSQQASHVRVTGGVDSILGPSAPA----------------P--TVSSP------SHIP    249
SnIMC15      HAPQHPPLAVHIYGGLESLVAAVALSLLGNSNIGLLGNLFCLPATAKKKESTTPQLNRAP    471
              : .:    *:: **::*::.  * :                *  : ..       .: *

TgIMC15      AFSQSSCGSTNSAPGNGQS-------------------------TWCQTSPVTVGGQANA    284
SnIMC15      ALKEAA----FSSPGKERRKSRDAGPPCLSGTTSRMTTASSMSPFSCSSPPCNSSGSSTP    527
             *:.:::     *:**: :                         *.: * . .*.:.

TgIMC15      SA-QVKI--AQAPAAASSQPNYSRAKTTQAVVEQKMVPEGYDRVEHMVPRLRPVSVVENR    341
SnIMC15      PSFHHRQASSWSPRRGLGAP-VAATGQSGVVENQLLRNEGYEKHEVIVPLLRPVKIVEKQ    586
              : : :   : :*  . . *  : :  : .* :* :  ***:: * :** ****.:**::

TgIMC15      IEIPVVKVVDTIVHKQKIEERIRFVDKPVVQEVEKFVEVPEVIYSDVIVEVPEIVEVIKH    401
SnIMC15      VEVPLIKRVDTVVPAARIEEKITFIDKPIVQQVERFVEVPEVHYTDVIVEVPEVIHVEKR    646
             :*:*::* ***:*   :***:* *:***:**:**:******* *:*********::.* *:

TgIMC15      VPKEEIRENITYVPRFETKIIPKYVDVPIIKIVDRYEEVHEIHEVLKPVAKVKVVDVPKE    461
SnIMC15      VPKEKIVENVIFVPRFEEKLIPKYVEVPVVKIVDRYEEVQQTEEVLKLVPKVKVVEVPRE    706
             ****:* **: :***** *:*****:**::*********:: .**** * *****:**:*

TgIMC15      FTRIVPKYSVHKTKSKVSVPDIQYKEVPRYRMRYEPKILRDTVINHIPQYLDIEVPYEVP    521
SnIMC15      VLRVVPKYVVHKTTKEIPVPVLQHKPVPRYKMRYEPKIIQQGVEKVIPQIKDVEIPYPVP    766
             . *:**** ****..:: ** :*:* ****:*******::: * : ***   *:*:** **

TgIMC15      KVTVVDQPFFVTTYRDHQYPVPVSHSVTPVLLPGDHTQVVDVPVEKPYVVVHDKFTPR-P    580
SnIMC15      VVKEVPAPYFVEKYRDRPVAVPVSHLVLPVFKEGSHAQQVDVAVPTPVLLVHDQLQPMAR    826
              *. *   *:** .***:    ***** * **:  *.*:* *** *  .* ::***:: *

TgIMC15      PIPELIPGQAVLRGVTHLDKERLTAEQQEQLARQTTPSRPASPDHRFWEREREAELRAPL    640
SnIMC15      PVPQLLAGAAAATA-QPLDPRCLNPEQRGS---------PATPM--LF------------    862
             *:*:*: * *.  .    ** . *. **: .          **:*   ::

TgIMC15      SPVHIRPPRQRKFFAGMPRQSCGPMPSSVDAQCCQSPWTQHVQQTNTLSYPN   692
SnIMC15      ---------------------------------------------------           862
```
