# Supplementary material for: Differential Roles for Inner Membrane Complex Proteins across Toxoplasma gondii and Sarcocystis neurona Development
Source: mSphere. 2017 Oct 18;2(5):e00409-17. doi: 10.1128/mSphere.00409-17 (PMC5646244; doi:10.1128/mSphere.00409-17)
Supplement: TABLE S1 [file sph005172388st10.pdf]

Table S1

#	Protospacer name	Sequence
1.	5-IMC12dKO-s	/5Phos/AAGTTGTACTTCGCTCTGTGAATTTGG
2.	5-IMC12dKO-as	/5Phos/AAAACCAAATTCACAGAGCGAAGTACA
3.	3-IMC12dKO-s	/5Phos/AAGTTGCGTCGACTCCATGCCCCAGTG
4.	3-IMC12dKO-as	/5Phos/AAAACACTGGGGCATGGAGTCGACGCA
5.	5-IMC14dKO-s	/5Phos/AAGTTGtaccaatagtggacacgATGG
6.	5-IMC14dKO-as	/5Phos/AAAACCATcgtgtccactattggtaCA
7.	3-IMC14dKO-s	/5Phos/AAGTTGAGTCCGCAGTCTCGTGAATTG
8.	3-IMC14dKO-as	/5Phos/AAAACAATTCACGAGACTGCGGACTCA
9.	5-IMC15dKO-s	/5Phos/AAGTTGAGAGAATTTTTCTAAAATGG
10.	5-IMC15dKO-as	/5Phos/AAAACCATTTTAGAAAAATTCTCTCA
11.	3-IMC15dKO-s	/5Phos/AAGTTGCACAGTGTTGCCAGTCACCGG
12.	3-IMC15dKO-as	/5Phos/AAAACCGGTGACTGGCAACACTGTGCA

#	IMC Primer name	Sequence
1.	5'IMC7-F-B1	GGGGACAAGTTTGTACAAAAAAGCAGGCTGCGTCCAGTTCACAGCTCCCAACAC
2.	5'IMC7-F-B4	GGGGACAACTTTGTATAGAAAAGTTGGGTGGCACTGGCAGAACGGCGTCTG
3.	3'IMC7-F-B3	GGGGACAACTTTGTATAATAAAGTTGcgATGGAGTTCACTGCTGACAACGTAC
4.	3'IMC7-R-B2	GGGGACCACTTTGTACAAGAAAGCTGGGTACGCGATCGTGAATGGTCGGAC
5.	5'IMC7ver-F	GAACGACCAGACATCCGATGTTCT
6.	3'IMC7ver-R	CGTGTACACCAAGTCCAAGGGC
7.	5'U6_sequence-F	ctcgtagagaacaagcactcg
8.	5'IMC12-DHFR-F	cactcctttatttgggaaaccaccttgtctttgctCACGAAACCTTGCATTCAAAC
9.	3'IMC12-DHFR-R	ctggtccacacttcccctctacggaaaagcggttcATCCTGCAAGTGCATAGAAGG
10.	5'IMC12ver-F	cagGTTTAAACCACCAACAAATCCAACAACTTCCG
11.	3'DHFR-CXR	ACTGCGAACAGCAGCAAGATCG
12.	5'DHFR-ver-F	CACACAGTCTCACCTCGCCT
13.	3'IMC12ver-R	cgtactacagcgtgttcacg
14.	IMC12dko-int-ver-F	GGGGACAAGTTTGTACAAAAAAGCAGGCTGCCAGCATGCTGGCGGTACCCAAC
15.	IMC12dko-int-ver-R	GGGGACAACTTTGTATAGAAAAGTTGGGTGCGCACAACCGGTGAACAGAATAC
16.	IMC12-S249A-S250A-R	CTCGCTCACGCGAGAGAAAACCCGCGGTG**CC**G**C**GGCGAGAGAGCCCACTGCCGCCTC
17.	IMC12-S249E-S250E-R	CTCGCTCACGCGAGAGAAAACCCGCGG**CTCCTC**GGCGAGAGAGCCCACTGCCGCCTC
18.	5'IMC14-DHFR-F	cttgtgtttcgtgtggaccggaaggaaatcCACGAAACCTTGCATTCAAACC
19.	3'IMC14-DHFR-R	catacggcatctgtatttgaacatactcgcctgcATCCTGCAAGTGCATAGAAGG
20.	5'IMC14ver-F	cagGTTTAAACTCGCATGCTGAGGAACCAACC
21.	3'IMC14ver-R	cgccgctatattaagccttgc
22.	IMC14dko-int-ver-F	gtgactgggcgatgagcg
23.	IMC14dko-int-ver-R	CGGGACATCACGGTACTTATACTC
24.	5'IMC15-DHFR-F	gaaaggacacgctttgcacacaccggcgtcacagcCACGAAACCTTGCATTCAAACC
25.	3'IMC15-DHFR-R	cgtttcctagacaatccgtctcctcaaggaggagcATCCTGCAAGTGCATAGAAGG
26.	5'IMC15ver-F	GTGTACTGGTGTTCGAGGCTATG
27.	3'IMC15ver-R	CAAACCAAATGATGCGGTCCCTG
28.	IMC15dko-int-ver-F	cagaactgcaagagacaccg
29.	IMC15dko-int-ver-R	ctgcagtgcaagaattccgc
